# Sexual and Reproductive Healthcare Among Youth With Autism and ADHD: A Population‐Based Study From Sweden

**DOI:** 10.1002/aur.70306

**Published:** 2026-07-28

**Authors:** Lovisa Hellsten, Jessica Rast, Anna Nielsen, Anna Mia Ekström, Kristina Gemzell‐Danielsson, Kyriaki Kosidou, Viktor H. Ahlqvist

**Affiliations:** ^1^ Department of Global Public Health Karolinska Institutet Stockholm Sweden; ^2^ Centre for Epidemiology and Community Medicine Region Stockholm Stockholm Sweden; ^3^ A.J. Drexel Autism Institute, Drexel University Philadelphia Pennsylvania USA; ^4^ Department of Emergency Medicine Denver Health and Hospital Authority Denver Colorado USA; ^5^ Department of Clinical Science and Education, Södersjukhuset Karolinska Institutet Stockholm Sweden; ^6^ Department of Infectious Diseases/Venhälsan Södersjukhuset Stockholm Sweden; ^7^ Department of Women's and Children's Health Karolinska Institutet Stockholm Sweden; ^8^ WHO Centre, Division of Gynecology and Reproductive Medicine Karolinska University Hospital Stockholm Sweden; ^9^ Department of Biomedicine Aarhus University Aarhus Denmark; ^10^ Institute of Environmental Medicine Karolinska Institutet Stockholm Sweden

**Keywords:** adolescent health, adolescent health services, attention deficit disorder with hyperactivity, autism spectrum disorder, health services accessibility, health services for persons with disabilities, preventive health services, reproductive health, sexual health, telemedicine

## Abstract

Youth with autism spectrum disorder or attention‐deficit/hyperactivity disorder (ADHD) may face barriers to sexual and reproductive health (SRH) services. Yet, data on preventive SRH service use remain limited, especially digital care. This population‐based registry study investigated whether patterns of SRH service use differed by digital versus in‐person care among 12–22‐year‐olds in Stockholm County (2018–2022). Among 454,405 youth (48.6% female; mean age 16 years), 11,850 were autistic, 31,144 had ADHD, and 9126 had both conditions; the majority of SRH contacts were in‐person visits. ADHD youth had higher adjusted Odds Ratios [aOR] of in‐person (females: 2.02 [95% CI, 1.93–2.11]; males, 1.46 [1.39–1.54]) and digital visits (females, 1.21 [1.14–1.29]; males, 1.26 [1.05–1.52]) than control peers. Autistic youth had lower aOR of in‐person (females, 0.67 [0.63–0.72]; males, 0.45 [0.40–0.51]) and digital visits (females, 0.79 [0.71–0.89]; males, 0.79 [0.54–1.15]). Intensity of use (incidence rate ratios) was higher among ADHD youth but similar among autistic females compared with control peers. Autistic females were more likely to consult physicians but less likely to see midwives or use short‐acting contraception. ADHD was associated with higher odds of all provider types, contraceptive methods, and abortion. In conclusion, ADHD youth engaged more and autistic youth less—particularly males—with SRH services than controls. Smaller relative digital than in‐person differences suggest digital options might reduce access disparities. Similar intensity of use between autistic and control females suggests barriers may be entry to care, whereas high SRH use among ADHD youth challenges assumptions that low engagement drives risk outcomes.

## Summary

Sexual and reproductive health services help support young people's health and wellbeing, but neurodiverse youth may face barriers to accessing care compared to their neurotypical peers. This study examined the use of sexual and reproductive health services among autistic and ADHD youth, including digital services. We found that young people with ADHD used services more than their peers, whereas autistic youth—particularly males—used them less often. Smaller differences in digital service use suggest that online pathways might help reduce inequalities in access to care.

## 1 Introduction

Adolescents with autism spectrum disorder (hereafter autistic individuals) or attention‐deficit/hyperactivity disorder (ADHD) have distinct needs pertaining to healthcare provision, including ability to process information, preferred communication style, and sensitivity to sensory or cognitive overstimulation (Bisset et al. 2023; Malik‐Soni et al. 2022). Sexual and reproductive health (SRH) services—including contraceptive counseling, sexually transmitted infection (STI) prevention, and support for bodily autonomy—are especially important during adolescence (Starrs et al. 2018), yet little is known about how neurodivergent youth engage with these services compared to their neurotypical peers.

ADHD symptoms may be associated with increased sexual risk‐taking, including earlier sexual debut, more sexual partners, higher rates of STIs, issues with adherence to oral contraceptives, and teenage pregnancies (Skoglund et al. 2019; Wallin, Alehagen, et al. 2024; White and Buehler 2012). For autistic individuals, healthcare systems frequently lack adaptations to meet needs like sensory sensitivities, communication differences, and executive functioning difficulties, compounded by stigma, insufficient provider training, and limited access to appropriate sex education (de Visser et al. 2025; Holmes et al. 2023; Sharma et al. 2024). Autistic females also show higher rates of menstrual and hormonal disorders as well as increased menstrual discomfort (Ames et al. 2024). Sexual victimization has additionally been found more prevalent among autistic and ADHD individuals, and among those with a co‐occurrence of both conditions (Motamed et al. 2025; White and Buehler 2012).

Digital modes of healthcare contact have been suggested as a means of providing autism‐aware care by reducing barriers including social and sensory stress, and meeting communication preferences (de Visser et al. 2025; Harris et al. 2022; Holmes et al. 2023; Motamed et al. 2025). For individuals diagnosed with ADHD, multimodal information dissemination has similarly been proposed as a facilitating factor for improved information retention (Klint Carlander et al. 2022; Wallin, Wallin‐Lundell, et al. 2024). Digital channels could, for instance, enable information delivery in “chunks” and the use of visual aids to overcome attention difficulties and distraction that impact ability to focus (Klint Carlander et al. 2022; Kumaresan et al. 2022; Lui et al. 2025). Yet, the role of digital modes of contact in providing SRH services for autistic and/or ADHD individuals remains underexplored (Lakes et al. 2022; Lamash et al. 2023).

Leveraging unique population‐based data from Stockholm County, this study examines SRH service utilization at Swedish Youth Clinics among autistic and/or ADHD adolescents and young adults aged 12–22. We assess differences in overall access, evaluate whether digital modes of contact may mitigate disparities in service use, investigate variation in the intensity of service use, and explore the content of care—specifically, visits with midwives versus physicians and, among females, the type of contraceptives dispensed, including short‐acting and long‐acting methods and abortion.

## 2 Methods

This study is reported according to the Strengthening the Reporting of Observational Studies in Epidemiology guidelines (Table S1).

### 2.1 Study Population

Using pseudonymized Swedish personal identification numbers, assigned to each resident upon birth or immigration, we linked several regional healthcare and administrative registries to create an open cohort of all individuals aged 12–22 residing in the Stockholm region between January 1, 2018, and December 31, 2022 (Figure S1). This resulted in 454,405 unique individuals eligible for inclusion in the analysis. No exclusions were made at the population level.

### 2.2 Youth Clinics and Sexual and Reproductive Healthcare Use

Since the 1970s, Swedish youth clinics have provided confidential, free‐of‐charge SRH services for adolescents and young adults. While young people may seek SRH care from other providers, youth clinics are the primary and most widespread SRH service setting for this age group in Stockholm and Sweden; this study therefore focuses on preventive, health‐promoting, and treatment‐oriented services delivered at these clinics.

In Stockholm, 31 clinics serve individuals aged 12–22, offering contraceptive counseling and provision (prescription/insertion), STI testing, and free emergency contraception and condoms, alongside psychosocial support from trained counselors (Zettergren et al. 2024). SRH services are primarily delivered by midwives, with physicians involved in more complex cases. Visits may be in‐person or digital, with asynchronous chat messaging and synchronous video consultations expanding since April 2020 (Hellsten et al. 2025).

For the purpose of this study, SRH use included all appointments with medical staff (midwives and physicians) at youth clinics from the Stockholm Regional Healthcare Data Warehouse (VAL) (Table S2) (Rajan et al. 2021). We excluded no‐shows, atypical contacts (e.g., care provided without patient involvement), and non‐digital remote contacts (e.g., telephone), due to inconsistent data coverage (Figure S2). The final analytic sample included 492,762 consultations involving medical staff (Tables S3 and S4). SRH use was categorized into in‐person and digital visits, with digital visits also being further sub‐categorized into video‐ and chat‐based consultations. The information on chat‐based consultations was extracted from the electronic medical record system “TakeCare”(Cars et al. 2013), which captures all publicly funded SRH services; chat data from three private clinics was not available.

In addition, we were able to obtain data on contraceptive use and abortions for all females aged 15–22 born 1996–2007 (*n* = 174,870) using linked prescription and clinical data from the VAL register (Table S5). Contraceptive use was defined as dispensation of a contraceptive at a pharmacy throughout the study period, categorized into having collected a prescription of a LARC method (e.g., hormonal/copper IUDs, implants) and/or short‐acting reversible contraceptive (SARC e.g., oral contraceptives, vaginal contraceptive ring, patch, and injectables). Information on abortion was dichotomized into a binary variable of having had an abortion (> = 1) over the study period. The provision of contraceptive and abortion services was not restricted to data from Youth Clinics, but included all healthcare providers in the Stockholm County offering the services between 2018 and 2022.

### 2.3 Autism and ADHD

Autistic and ADHD youth were primarily identified using the International Classification of Diseases, 10th Revision (ICD‐10), from regional administrative healthcare records covering specialist, in‐patient, outpatient, and primary care (ICD‐10: F84 & F90, respectively). For ADHD, diagnostic data were supplemented with prescription records of centrally acting sympathomimetics (Anatomical Therapeutic Chemical (ATC) code N06BA), obtained from a regional administrative healthcare register (VAL‐LAK, Table S2) including all dispensed medications covered by the pharmaceutical benefits scheme.

Diagnoses and prescriptions were traced 5 years prior to each study year. If identified within that window, individuals were classified with autism or ADHD for all study years, using a binary indicator variable for each condition. We matched these diagnosed individuals to peers without a formal diagnosis, henceforth referred to as ‘control peers’, recognizing that these individuals will mostly never be diagnosed—but could also include some individuals who will later be diagnosed or have the underlying condition but never be diagnosed. This resulted in four mutually exclusive groups: autism, ADHD, both autism and ADHD, and control peers.

### 2.4 Sociodemographic Characteristics

The primary covariates included legal sex, birth year quintiles, migration status (Swedish‐born parents, foreign‐born parents, and foreign‐born individuals), the highest level of parental education (primary education, secondary education, or university level), and birth‐year‐specific quintiles of family disposable income recorded between 2018 and 2020. Parental linkages, sociodemographic information, and country of birth were sourced from registries maintained by Statistics Sweden (Ekbom 2011; Ludvigsson et al. 2019; Willson and Shuey 2019). Detailed descriptions of each parameter can be found in Table S2. Information on parental education and/or family income was missing for 97,647 individuals (21.5%), primarily due to incomplete or outdated parental linkage data. These individuals were retained in the main analysis using a missing‐indicator approach (Song et al. 2021).

### 2.5 Statistical Analysis

All analyses were stratified by sex and estimated across the mutually exclusive diagnostic groups given marked differences in diagnostic prevalence (Bolte et al. 2023; Dimitri et al. 2025; Napolitano et al. 2022) and SRH service use between males and females (Hellsten et al. 2025).

First, we described the study population using counts and percentages for categorical variables and means with standard deviations for continuous variables. Second, we estimated odds ratios (ORs) and 95% confidence intervals (CIs) for any youth clinic SRH service use by mode of contact during the study period, using multivariable logistic regression analysis. Differences between fully adjusted in‐person and digital models were tested using *χ* ^2^ tests based on seemingly unrelated estimation. Third, we estimated the incidence rate ratio (IRR) of the number of visits over the study period (count of visits across the entire study period) using a zero‐inflated Poisson regression, using a constant‐only inflation equation, assuming a uniform probability of excess zeros across all observations. Finally, we estimated ORs and 95% CIs for having visits with a midwife or physician, and, among females, for any contraceptive use as well as for use of LARC and SARC contraceptive methods, using multivariable logistic regression analysis.

To enhance clinical and public health interpretability, we report both relative measures (ORs or IRRs) comparing autistic and/or ADHD adolescents and young adults with control peers, as well as covariate‐adjusted absolute measures—study period prevalence for binary outcomes and rates per 1000 person‐years for count outcomes. Model I was adjusted for birth year quintiles (1996–1999, 2000–2002, 2003–2005, 2006–2008, 2009–2010) whereas model II was adjusted for birth year quintiles and sociodemographic predictors (household disposable income, parental education, and migration background). We reported on the adjusted estimates in the main manuscript. All analyses and visualizations were conducted in Stata version 18, using *α* = 0.05.

### 2.6 Sensitivity Analysis

As sensitivity analysis, we repeated the main analysis restricted to complete cases to check for consistency. We also repeated the main models adjusting for birth year as a continuous variable, including both linear and squared terms to account for potential non‐linear effects. Finally, as the primary analysis relied on mutually exclusive diagnostic groups, we also analyzed autistic and ADHD groups individually (i.e., overlapping diagnostic groups). For this analysis, three models were specified: Model I adjusted for birth year (grouped into quintiles); Model II for birth year and comorbidity (i.e., both conditions are jointly in the model); and Model III adjusted for the remaining sociodemographic predictors (household disposable income, parental education, migration background, and birth region).

## 3 Results

### 3.1 Descriptive Characteristics

Among the 454,405 individuals aged 12–22 included in this study between 2018 and 2022 (48.6% female; Table 1), the majority were Swedish born, had Swedish born parents, and had parents with secondary or post‐secondary education. Autism and ADHD were more prevalent among males than females: autism (3.0% vs. 2.2%), ADHD (7.7% vs. 6.0%), and co‐occurring conditions (2.5% vs. 1.5%). Diagnoses were more common among youth in the lower three quintiles of household income compared to those in the highest two quintiles.

**TABLE 1:** Sociodemographic characteristics of the study population by sex and autism and/or ADHD among youth aged 12–22 in Stockholm County, 2018–2022.

	Females *N* = 220,729 (48.6%)				Males *N* = 233,676 (51.4%)			
	Control peers 199,228 (90.3%)	Autism 4899 (2.2%)	ADHD 13249 (6.0%)	Autism & ADHD 3353 (1.5%)	Control peers 203,057 (86.9%)	Autism 6951 (3.0%)	ADHD 17895 (7.7%)	Autism & ADHD 5773 (2.5%)
Age category^a^, *N* (% of person‐years)^a^								
12–14	85,253 (12.9)	1938 (10.3)	4242 (8.1)	982 (7.2)	84,461 (12.4)	3583 (14.3)	7632 (11.2)	2433 (10.9)
15–19	295,303 (44.7)	9704 (51.8)	26,420 (50.5)	7347 (53.7)	299,921 (43.9)	12,181 (48.8)	34,818 (50.9)	11,590 (51.7)
20–22	280,191 (42.4)	7086 (37.8)	21,655 (41.4)	5344 (39.1)	298,200 (43.7)	9216 (36.9)	25,909 (37.9)	8377 (37.4)
Migration background, *N* (%)								
Swedish‐born parents	124,890 (62.7)	3929 (80.2)	10,625 (80.2)	2882 (86.0)	123,765 (61.0)	5049 (72.6)	14,016 (78.3)	4821 (83.5)
Foreign‐born parents	30,666 (15.4)	549 (11.2)	1664 (12.6)	258 (7.7)	30,125 (14.8)	1202 (17.3)	2410 (13.5)	590 (10.2)
Foreign‐born	43,672 (21.9)	421 (8.6)	960 (7.2)	213 (6.4)	49,167 (24.2)	700 (10.1)	1469 (8.2)	362 (6.3)
Maternal education level, *N* (%)								
Primary education	46,874 (23.5)	1116 (22.8)	3732 (28.2)	845 (25.2)	45,695 (22.5)	1690 (24.3)	5157 (28.8)	1493 (25.9)
Secondary education	62,131 (31.2)	1708 (34.9)	5014 (37.8)	1276 (38.1)	60,998 (30.0)	2436 (35.0)	6617 (37.0)	2061 (35.7)
Post‐secondary education	74,222 (37.3)	2004 (40.9)	4335 (32.7)	1208 (36.0)	74,638 (36.8)	2692 (38.7)	5906 (33.0)	2168 (37.6)
Unknown	16,001 (8)	71 (1.4)	168 (1.3)	24 (0.7)	21,726 (10.7)	133 (1.9)	215 (1.2)	51 (0.9)
Paternal education level, *N* (%)								
Primary education	57,761 (29)	1552 (31.7)	5428 (41.0)	1271 (37.9)	56,729 (27.9)	2170 (31.2)	7268 (40.6)	2186 (37.9)
Secondary education	60,171 (30.2)	1714 (35.0)	4428 (33.4)	1167 (34.8)	59,608 (29.4)	2336 (33.6)	6003 (33.5)	1973 (34.2)
Post‐secondary education	57,768 (29)	1392 (28.4)	2789 (21.1)	784 (23.4)	58,274 (28.7)	2055 (29.6)	3816 (21.3)	1379 (23.9)
Unknown	23,528 (11.8)	241 (4.9)	604 (4.6)	131 (3.9)	28,446 (14.0)	390 (5.6)	808 (4.5)	235 (4.1)
Household income, *N* (%)								
Q1	37,290 (18.7)	918 (18.7)	2508 (18.9)	646 (19.3)	36,636 (18.0)	1532 (22.0)	3390 (18.9)	1096 (19.0)
Q2	32,680 (16.4)	1135 (23.2)	3090 (23.3)	845 (25.2)	32,426 (16.0)	1587 (22.8)	4037 (22.6)	1411 (24.4)
Q3	32,588 (16.4)	1091 (22.3)	2668 (20.1)	732 (21.8)	32,613 (16.1)	1308 (18.8)	3650 (20.4)	1229 (21.3)
Q4	33,779 (17)	818 (16.7)	2324 (17.5)	576 (17.2)	33,762 (16.6)	1047 (15.1)	2943 (16.4)	948 (16.4)
Q5	34,695 (17.4)	612 (12.5)	2075 (15.7)	433 (12.9)	34,617 (17.0)	770 (11.1)	2711 (15.1)	732 (12.7)
Unknown	28,196 (14.2)	325 (6.6)	584 (4.4)	121 (3.6)	33,003 (16.3)	707 (10.2)	1164 (6.5)	357 (6.2)

^a^ Age categories are based on person‐years during follow‐up (2018–2022); individuals may contribute to multiple age categories.

Over the study period, 492,762 SRH youth clinic visits (90.1% female) were recorded, amounting to approximately 100,000 visits annually (Table S3). The majority (93.7%) of contacts were in‐person visits, with digital modalities accounting for a minority of overall SRH services across all groups (Table S4). Over the study period, overall SRH service use (> = 1 visit) was significantly higher in the female (39.5%) than male population (9.3%) across all modes of contact (Table S6). Midwives provided the vast majority of youth clinic SRH care (88.6% of visits), while fewer than 10% of visits involved a physician.

### 3.2 SRH Service Use and Intensity

**FIGURE 1:**
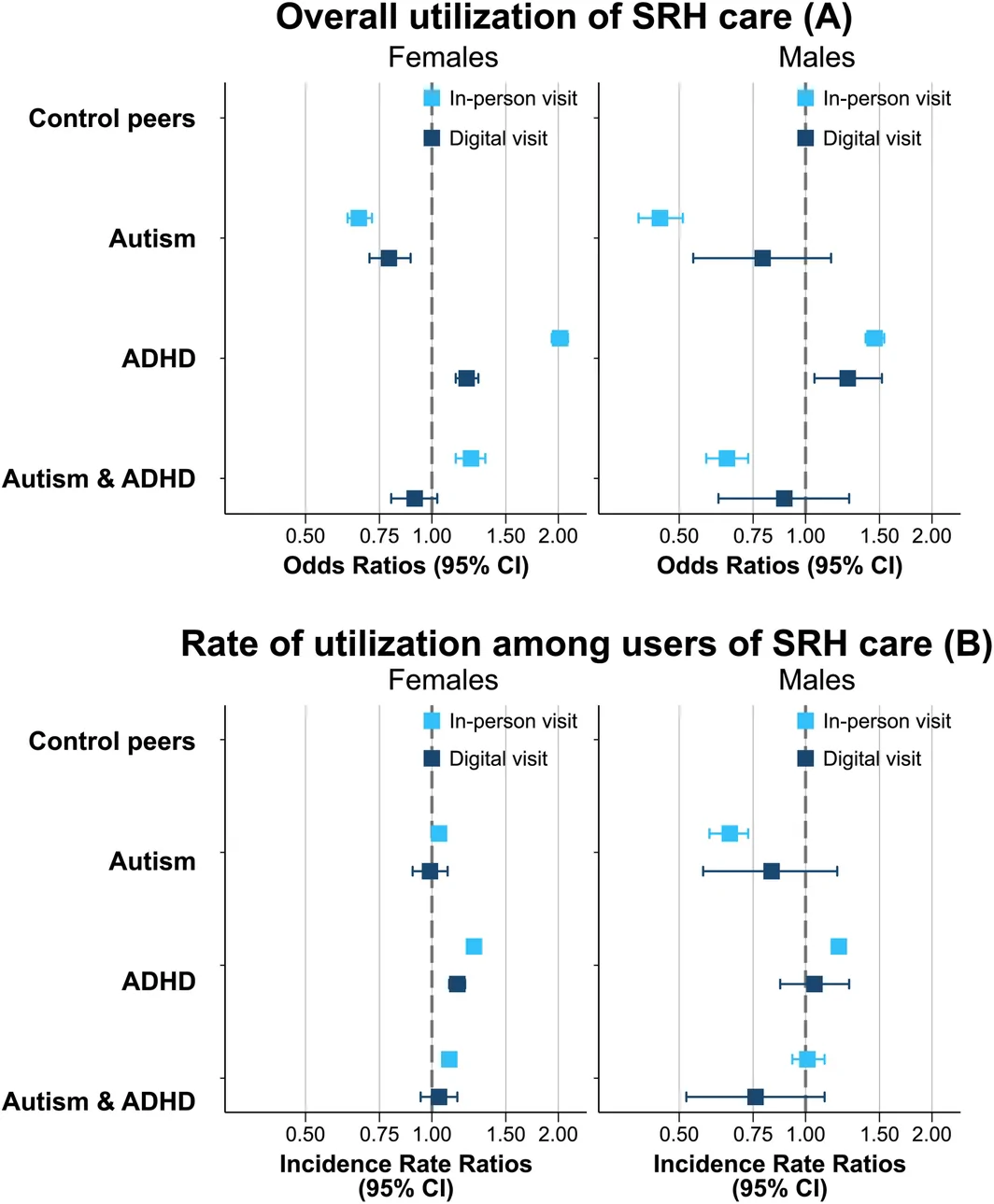
Adjusted odds ratios (OR) and 95% CIs of sexual and reproductive health (SRH) service use at the population level for in‐person and digital visits (A) adjusted Incidence rate ratios (IRR) and 95% CIs of intensity of SRH service use for in‐person and digital visits (B). Estimates are stratified by sex among autistic and/or ADHD youth compared to control peers for the entire study period 2018–2022. Model adjusted for birth year, migrant background, parental education, and household income. Error bars represent 95% CI.

Patterns of SRH service use differed notably across the autistic and ADHD population, for overall youth‐clinic visits (in‐person and digital) and contraceptives and abortions delivered by any healthcare provider. Among both males and females, adolescents and young adults with ADHD consistently demonstrated higher use of services across all modes of contact compared with their control peers (Figure 1, panel A; Tables S7–S9). The relative differences between control peers and autistic and/or ADHD youth were, however, more pronounced for in‐person visits than for digital visits. In contrast, autistic individuals had fewer contacts across all modalities, encompassing both in‐person visits and digital visits (Figure 1, panel A; Tables S7–S9).

When digital visits were further separated into video and chat consultations, wide confidence intervals reflected considerable uncertainty in the contact‐specific estimates across all groups (Tables S10 and S11). Autistic females (*p* for difference between physical and digital visits = 0.007) and males (*p* = 0.004) showed usage patterns more similar to their control peers for digital than for in‐person visits (Table S12). In contrast, females with ADHD (*p* < 0.001) and, to a lesser extent, males (*p* = 0.125) were more likely to use in‐person rather than digital services, although the estimates for males were imprecise.

Those with co‐occurring autism and ADHD generally exhibited intermediate patterns of use—greater than autistic youth but lower than ADHD youth (Figure 1, Panel A; Tables S7–S9). Among females with co‐occurring autism and ADHD, in‐person visits at youth clinics were more likely than among control peers. Among males, however, this group had lower use than their undiagnosed counterparts.

Analyses yielded similar patterns for intensity of use across both in‐person and digital visits (Figure 1, Panel B; Tables S13 and S14). ADHD was associated with a higher frequency of overall youth clinic visits across the study period across both sexes (Table S15). Among autistic youth, intensity of use was similar to control peers among females but not males. Youth with co‐occurring autism and ADHD again fell between the two single‐diagnosis groups among females, with intensity of SRH service use for overall visits being comparable to that of control peers (Table S15).

### 3.3 Healthcare Providers, Female Contraceptive Use, and Abortion

Patterns by type of healthcare provider mirrored those observed for overall SRH service use (Table 2; Tables S16 and S17). Both ADHD females and males were more likely to have youth clinic visits with midwives and physicians compared to control peers. However, among autistic females, an exception emerged: they were more likely than control peers to have seen a physician (aOR: 1.24; 95% CI: 1.14 to 1.35), but less likely to have seen a midwife (aOR: 0.64; 95% CI: 0.60 to 0.69). In contrast, autistic males were less likely to have had visits with either healthcare provider than their undiagnosed control peers.

**TABLE 2:** Adjusted^a^ period prevalence, odds ratios, and 95% confidence intervals, of having had a SRH visit with a midwife or physician in the entire study population (*N* = 454,405), 2018–2022.

	Visits with midwife *n* = 105,285 (23.2%)		Visits with physician *n* = 29,386 (6.5%)	
	Period prevalence (95% CI)	Odds ratio (95% CI)	Period prevalence (95% CI)	Odds ratio (95% CI)
Females				
Control peers	32.9 (32.6, 33.2)	1.00	10.6 (10.4, 10.8)	1.00
Autism	25.1 (24.0, 26.3)	0.64 (0.60, 0.69)	12.8 (11.9, 13.7)	1.24 (1.14, 1.35)
ADHD	46.7 (45.8, 47.6)	2.02 (1.94, 2.11)	16.4 (15.8, 17.1)	1.68 (1.60, 1.76)
Autism & ADHD	36.4 (34.9, 38.0)	1.21 (1.11, 1.31)	15.8 (14.7, 17.0)	1.60 (1.46, 1.75)
Males				
Control peers	5.5 (5.3, 5.7)	1.00	1.3 (1.2, 1.4)	1.00
Autism	2.5 (2.2, 2.9)	0.44 (0.38, 0.50)	0.9 (0.7, 1.1)	0.64 (0.51, 0.81)
ADHD	7.9 (7.5, 8.3)	1.49 (1.41, 1.56)	1.6 (1.4, 1.8)	1.23 (1.11, 1.37)
Autism & ADHD	3.6 (3.2, 4.0)	0.63 (0.56, 0.71)	0.9 (0.7, 1.2)	0.71 (0.56, 0.89)

^a^ Adjusted for birth year and sociodemographic predictors (migrant background, parental education, and household income).

Among *N* = 134,907 females aged 15–22 years, more than half (56.3%) had dispensed a contraceptive at some point during the study period, with short‐acting reversible contraception (SARC) being more common (48.2%) than long‐acting reversible contraception (LARC) (13.7%) (Table S5). In the same age group, 4.1% had at some point had an abortion.

**FIGURE 2:**
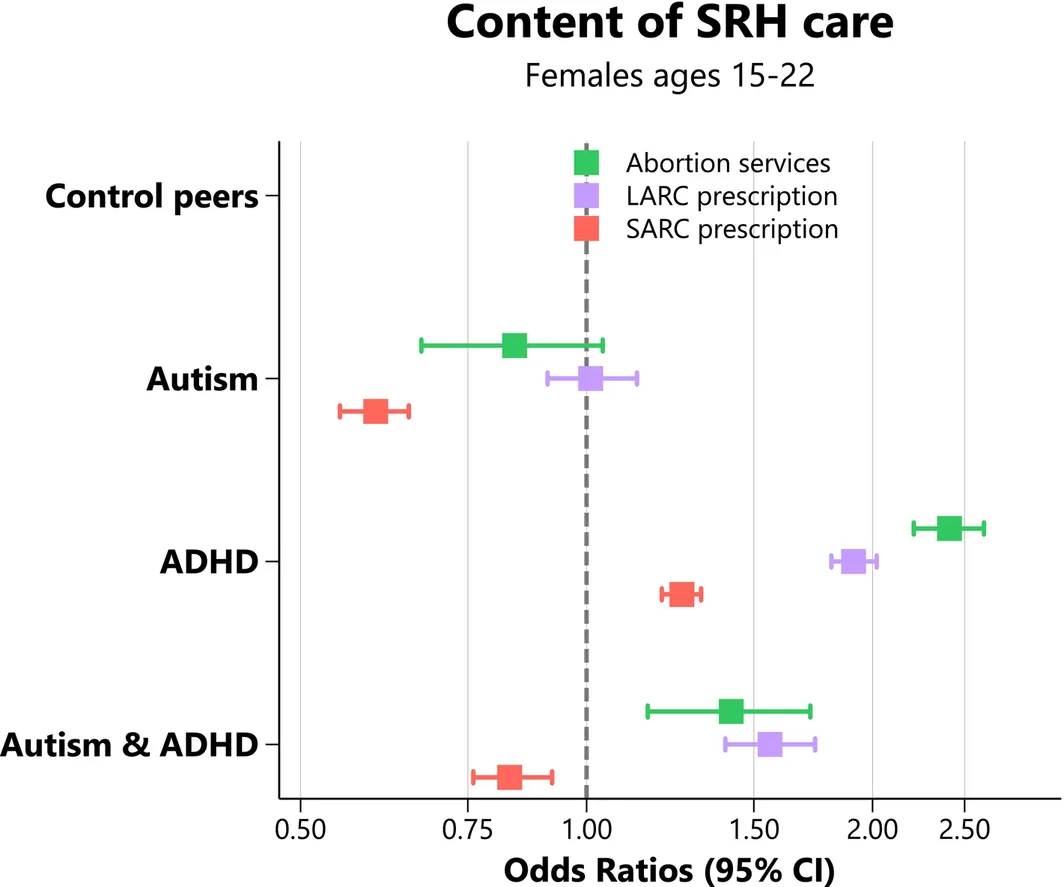
Adjusted odds ratios (OR) and 95% CIs content of care among females aged 15–22. Estimates compared to control peers for the entire study period 2018–2022. Estimates show prescriptions of long‐acting reversible contraceptives (LARC), short‐acting reversible contraceptives (SARC), and abortion services among autistic and/or ADHD youth compared to control peers for the entire study period 2018–2022. Model adjusted for birth year, migrant background, parental education, and household income. Error bars represent 95% CI.

The highest overall contraceptive uptake was observed among ADHD females, with more pronounced relative differences in uptake compared to their control peers seen for LARC (aOR: 1.91; 95% CI: 1.81 to 2.02) than SARC (aOR: 1.26; 95% CI: 1.20 to 1.32) (Figure 2; Table S18). In contrast, autistic females had lower SARC use (aOR: 0.60; 95% CI: 0.55 to 0.65) and lower overall contraceptive uptake, but were equally likely to have dispensed LARC as the control peers (aOR: 1.01; 95% CI: 0.91 to 1.13). Females with co‐occurring autism and ADHD showed higher overall uptake of LARC compared to their control peers, but not for SARC after adjusting for sociodemographic characteristics (Figure S3; Table S18).

The highest odds of having had an abortion (1 ≤) were observed among ADHD females (aOR: 2.41; 95% CI: 2.21 to 2.62) and among females with co‐occurring autism and ADHD diagnosis (aOR: 1.42; 95% CI: 1.16 to 1.72). Among autistic females, there was no significant difference compared to control peers (aOR: 0.84; 95% CI: 0.67 to 1.04) (Figure 2; Table S18).

### 3.4 Sensitivity Analysis

The sensitivity analyses were consistent with the main analyses, with no meaningful differences in direction or interpretation. Results from the models restricted to complete‐cases were comparable to the main analysis (Tables S19–S26), and although absolute prevalence was somewhat higher in models adjusting for linear and squared birth year terms, relative differences between control peers and autistic or ADHD youth remained consistent (Tables S27–S36). When analyzing autism and ADHD separately, adjusting for comorbid ADHD (Model II) shifted the direction of association for several outcomes for autistic youth; the adjusted odds of both in‐person and digital SRH visits changed from higher to lower compared with control peers (Tables S37–S47).

## 4 Discussion

In this population‐based study of Stockholm youth, individuals with ADHD consistently showed higher use of SRH services than peers without ADHD, across both sexes and all modes of care. Among ADHD females, contraceptive dispensation and abortion rates were higher than control peers. By contrast, autistic youth generally had lower SRH service use, except for consultations with physicians. Those with co‐occurring autism and ADHD showed intermediate patterns, with some sex‐specific differences. The majority of contacts were conducted in person, reflecting the initial phase of digital SRH service implementation and its early adopters. Differences in use were less pronounced for digital versus in‐person visits. For autistic females, intensity of use matched that of control peers, despite lower overall use.

### 4.1 Comparison to Existing Literature

The elevated use of services by ADHD youth aligns with prior evidence linking ADHD to greater sexual risk‐taking and increased rates of teenage pregnancy and other complications (Skoglund et al. 2019; Wallin, Alehagen, et al. 2024; White and Buehler 2012). Most earlier studies, however, have emphasized adverse outcomes and speculated that these arise from care failures including delayed or insufficient support combined with impulsivity and risk behaviors (Klint Carlander et al. 2022; Ostergaard et al. 2017; Rad et al. 2025; Skoglund et al. 2019; Wallin et al. 2022). Our findings provide evidence that ADHD youth, particularly females, in fact do engage more with SRH services—including contraceptives and abortion—suggesting that excess risks of teenage pregnancies and other complications might not be explained solely by underutilization of preventive care. Contraceptive method and compliance or inconsistency in use (e.g., higher degree of switching or discontinuation of SARC) may still influence effectiveness, regardless of dispensed prescriptions, warranting further research (Klint Carlander et al. 2022).

In our study, autistic youth showed lower use of SRH services overall. A similar pattern was observed in a US study where OB/GYN uptake was nearly 40% lower among autistic females (14–25 years) compared to the general population (Ames et al. 2021). The same study also found a lower IUD insertion rate among autistic youth compared to their peers, while we observed no marked differences for long‐acting methods but lower use of short‐acting contraceptives. Consistent with previous smaller‐scale studies (Ames et al. 2024), however, we found that young autistic females received fewer contraceptive prescriptions overall. Lower contraceptive use may reflect unmet care needs, as prior reports have shown that autistic females frequently experience menstruation‐related discomfort and gender dysphoria, often motivating interest in menstrual suppression (Lowik et al. 2025; Schwartz et al. 2022; Warrier et al. 2020; Weiselberg 2022).

In this study, autistic females were more likely than their control peers to have consulted a physician, but less likely to have visited a midwife. As physicians are consulted in more complex issues that exceed the responsibility of midwives, the difference may reflect contraceptive counseling for non‐anticonception reasons.

For ADHD males, the higher SRH service use observed is consistent with studies documenting greater risk of STIs, higher sexual activity, and poorer psychosexual functioning compared with control peers (Chen et al. 2018; Young and Cocallis 2023). In contrast, autistic males had the lowest service uptake, consistent with previous self‐reported studies indicating lower likelihood of partnered sex, fewer partners, and fewer sexual experiences than control peers and autistic females (Dewinter et al. 2016; May et al. 2017; Pecora et al. 2019).

Social interaction and communication represent key challenges for autistic individuals, and may influence the timing of partnered relationships as these skills are important for both romantic and sexual experiences (Parchomiuk 2019). Autistic females report more masking and camouflaging behavior and sexual experiences than males, which might influence SRH needs differently, despite small overall sex differences in sexual debut in Sweden (Cruz et al. 2024; Pecora et al. 2016; Public Health Agency of Sweden 2025). Lower SRH service use among autistic youth likely also reflects structural and interpersonal barriers—such as inadequate sex education and assumptions of asexuality—rather than lack of interest in romantic or sexual experience, potentially delaying relational “know‐how”(Graham Holmes et al. 2020).

Proportion of service use however only offers a partial view of utilization for both males and females. Despite lower odds of initiating SRH care, autistic females who did access youth clinics had an intensity of use comparable to control peers. This may suggest that barriers mostly lie in entry to care, rather than ongoing engagement, and points to possible unmet needs. In contrast, autistic males demonstrated persistently lower intensity of use. It could be that sensory sensitivities and communication challenges contribute to barriers in seeking care and the overall lower SRH service use observed among autistic youth (Motamed et al. 2025; Pellicano et al. 2022).

Finally, digital modes of SRH care have been suggested as a means to increase accessibility and reduce sensory stressors (de Visser et al. 2025; Harris et al. 2022; Holmes et al. 2023). In this study, the differences between autistic and/or ADHD youth and their control peers were somewhat smaller for digital than for in‐person visits. This might suggest that digital modes could mitigate some of the barriers to care. However, as digital care expands, its full impact requires evaluation; it remains unclear whether digital services will primarily increase the volume of contacts (i.e., healthcare consumption in some groups) or translate into improved SRH outcomes over time.

Lastly, while our findings suggest smaller group differences for digital visits, in‐person care dominated in this early phase of digital health implementation. Furthermore, while analyzed as separate outcomes here, digital and in‐person modalities are often integrated; such hybrid care models remain underexplored for SRH services but might offer promising future pathways.

### 4.2 Strengths & Weaknesses

A key strength of this study is the use of total population data, enabling examination of actual preventive SRH service utilization among autistic and/or ADHD youth compared with control peers. To our knowledge, this is the first empirical population‐based study to take a step beyond ‘adverse outcomes’ (e.g., teenage pregnancies) and evaluate outcomes across multiple dimensions, including mode of contact, provider type, contraceptive use, and abortion.

This study also has limitations. In our data, we lacked information on co‐occurring intellectual disability. Individuals with intellectual disability face barriers to healthcare utilization, with possible unmet SRH needs as repercussion (Krahn et al. 2006). Our findings therefore likely mask heterogeneity within the population, including for healthcare access. Furthermore, this study lacked information on hormonal and gynecological conditions that frequently co‐occur with autism and ADHD. These might further explain differences in proportions by appointment type (midwives or physicians) or contraceptive method (long/short acting).

We restricted follow‐up to individuals aged ≤ 22 years; our cohort therefore primarily reflects early‐life diagnoses and likely captures youth presenting with the most pronounced symptoms. While Swedish registries provide comprehensive coverage, reliance on ICD‐10 and ATC codes limits diagnostic validation and precludes assessment of symptom severity. Some individuals in the comparison group may therefore have undiagnosed conditions without yet having received a formal diagnosis. Previous research has observed that females tend to receive their formal autism or ADHD diagnosis at a later age (Dalsgaard et al. 2020; Loomes et al. 2017; Skoglund et al. 2024). This may translate into later access to supportive care and potentially impact healthcare use differently pre‐ versus post formal diagnosis (Prasad et al. 2023). As a result, our findings may underestimate associations between autism and/or ADHD with SRH service use.

Furthermore, while analyses were restricted to legal sex, gender minority identities are more common among autistic individuals than in the general population (Bonazzi et al. 2025; Kallitsounaki and Williams 2023). Gender minorities face significant barriers, such as stigma and discrimination, with implications for SRH service access and use (Allen et al. 2025; Starrs et al. 2018). The inability to account for gender identity and its link to SRH service use thus warrants further research.

The Swedish setting—a digitally mature society with universal SRH coverage—may also constrain generalizability, particularly to less urban or resource‐limited contexts or contexts with a lower degree of digital equality. Finally, as digital services represent a relatively recent development, available data still remain limited and warrant further study.

### 4.3 Implications

These findings provide a population‐level view of SRH service utilization among autistic and ADHD youth, revealing consistent differences from control peers. ADHD youth engage more with services, whereas autistic youth, particularly males, engage less. How these patterns align with underlying needs requires further study, but the results underscore the importance of tailoring SRH services for neurodiverse populations. Service planning should account for potential barriers to access among autistic youth, as well as the elevated demand and risk observed in ADHD individuals. Integrating awareness of neurodevelopmental conditions into SRH care delivery may improve equity and ensure services are responsive to the diverse needs of young people.

## 5 Conclusions

ADHD youth had higher use of SRH services than non‐diagnosed control peers, with females showing increased contraceptive dispensation and abortion rates. In contrast, autistic youth generally used SRH services less often, except for physician consultations. Differences appeared less pronounced for digital visits, albeit more data is needed. Among autistic females, comparable intensity of use despite lower overall engagement suggests potential unmet service needs.

## Author Contributions

All authors contributed to the conceptualization of the study, K.K. secured the funding, and V.H.A. and K.K. supervised the study. L.H. curated the data, performed the analysis and visualizations. L.H. drafted the original manuscript together with V.H.A. All authors critically revised the manuscript for important intellectual content and contributed to the development of the study methodology. L.H. had full access to all the data in the study and takes responsibility for the integrity of the data and the accuracy of the data analysis. All authors read and approved the final manuscript.

## Funding

This work was supported by ALF funding (Agreement concerning Research and Education of Doctors) provided through Region Stockholm and Karolinska Institutet (Grant nr FoUI‐962046 to Kyriaki Kosidou). The funders had no role in the study design, data collection and analysis, preparation of the manuscript, or decision to publish.

## Ethics Statement

This study was performed in accordance with relevant guidelines/regulations, with ethical approval granted by the Swedish Regional Ethics Board (DNR: 2021‐03075, 2023‐02929‐02, and 2024‐01680‐02).

## Conflicts of Interest

The authors declare no conflicts of interest.

## Supporting information


**Table S1:** STROBE Statement—Checklist of items that should be included in reports of cohort studies.
**Figure S1:** Annual populations and study population, Stockholm residents 2018–2022.
**Table S2:** Variable description and sources.
**Figure S2:** Flow‐chart of included contacts at youth clinics in Stockholm, 2018–2022.
**Table S3:** Trends in youth clinic *visits by year* and exclusive diagnosis groups, 2018–2022 (*K* = 492,762).
**Table S4:** Visits at Stockholm Youth Clinics by Diagnosis Group, Mode of Contact, and Healthcare Provider over the entire study period (*K* = 492,762).
**Table S5:** Proportion among females ages 15–22 with any contraceptive prescription, long‐acting reversible contraception, short‐acting reversible contraception, or abortion over the study period 2018–2022 (*N* = 174,870).
**Table S6:** Sociodemographic characteristics of *SRH service use* by sex and exclusive diagnosis groups among youth aged 12–22 in Stockholm County, 2018–2022.
**Table S7:** Period prevalence and Odds Ratios of *overall SRH visits* at the population level across exclusive diagnosis groups over the study period 2018–2022.
**Table S8:** Adjusted period prevalence and Odds Ratios of *in‐person SRH visits* at the population level across exclusive diagnosis groups over the study period 2018–2022.
**Table S9:** Adjusted period prevalence and Odds Ratios of *digital SRH visits* at the population level across exclusive diagnosis groups over the study period 2018–2022.
**Table S10:** Adjusted period prevalence and Odds Ratios of *video SRH visits* at the population level across exclusive diagnosis groups over the study period 2018–2022.
**Table S11:** Adjusted period prevalence and Odds Ratios of *chat SRH visits* at the population level across exclusive diagnosis groups over the study period 2018–2022.
**Table S12:** Adjusted Odds Ratios and cross‐model Comparisons (*χ* ^2^ Tests) of in‐person and digital SRH visits across exclusive diagnosis group and sex over the study period (2018–2022).
**Table S13:** Visits per 1000 individuals and Incident‐Rate Ratios (IRR) *of in‐person SRH visits* among youth with a youth clinic visit, stratified by sex and across exclusive diagnosis groups, over the study period (95% CI).
**Table S14:** Visits per 1000 individuals and Incident‐Rate Ratios (IRR) of *digital SRH visits* among youth with a youth clinic visit, stratified by sex and across exclusive diagnosis groups, over the study period (95% CI).
**Table S15:** Visits per 1000 individuals and Incident‐Rate Ratios (IRR) of *overall SRH visits* among youth with a youth clinic visit, stratified by sex and across exclusive diagnosis groups, over the study period (95% CI).
**Table S16:** Adjusted period prevalence and Odds Ratios of *SRH visits with midwives* at the population level across exclusive diagnosis groups over the study period 2018–2022.
**Table S17:** Adjusted period prevalence and Odds Ratios of *SRH visits with physicians* at the population level across exclusive diagnosis groups over the study period 2018–2022.
**Figure S3:** Unadjusted and adjusted odds ratios (OR) and 95% CIs of content of care among females aged 15–22 over the study period. Content shows abortion services and prescriptions of long‐acting reversible contraceptives (LARC) and short‐acting reversible contraceptives (SARC) among autistic and/or ADHD youth compared to non‐diagnosed control peers for the entire study period 2018–2022. Model 1 adjusted for birth year, Model 2 adjusted for birth year, migrant background, parental education, and household income. Error bars represent 95% CI.
**Table S18:** Adjusted period prevalence and Odds Ratios of females aged 15–22 having used *contraceptives* (any, LARC, SARC) or *abortion* over the study period 2018–2022 (*N* = 174,870).
**Table S19:** Adjusted period prevalence, odds ratios, and 95% confidence intervals, of *overall, in‐person, and digital SRH visits* across exclusive diagnosis groups, for females and males separately over the study period 2018–2022, using only complete cases (*N* = 356,758).
**Table S20:** Fully adjusted* period prevalence, odds ratios, and 95% confidence intervals, of *overall, in‐person, and digital SRH visits* across exclusive diagnosis groups, for females and males separately over the study period 2018–2022, using only complete cases (*N* = 356,758).
**Table S21:** Adjusted period prevalence and Odds Ratios of *SRH visits with midwives* at the population level across exclusive diagnosis groups over the study period 2018–2022, using only complete cases (*N* = 356,758).
**Table S22:** Adjusted period prevalence and Odds Ratios of *SRH visits with physicians* at the population level across exclusive diagnosis groups over the study period 2018–2022, using only complete cases (*N* = 356,758).
**Table S23:** Adjusted period prevalence and Odds Ratios of females aged 15–22 having used *contraceptives* (any, LARC, SARC) or *abortion* over the study period 2018–2022, using only complete cases (*N* = 110,216).
**Table S24:** Visits per 1000 individuals and Incident‐Rate Ratios (IRR) *of overall SRH visits* among youth with a youth clinic visit, stratified by sex and across exclusive diagnosis groups, over the study period using only complete cases (*N* = 356,758).
**Table S25:** Visits per 1000 individuals and Incident‐Rate Ratios (IRR) *of in‐person SRH visits* among youth with a youth clinic visit, stratified by sex and across exclusive diagnosis groups, over the study period using only complete cases (*N* = 356,758).
**Table S26:** Visits per 1000 individuals and Incident‐Rate Ratios (IRR) *of digital SRH visits* among youth with a youth clinic visit, stratified by sex and across exclusive diagnosis groups, over the study period using only complete cases (*N* = 356,758).
**Table S27:** Birth year and birth year squared adjusted odds ratios, and 95% confidence intervals, of *overall, in‐person, and digital SRH visits* across exclusive diagnosis groups, for females and males separately over the study period 2018–2022.
**Table S28:** Fully adjusted* odds ratios, and 95% confidence intervals, of *overall, in‐person, and digital SRH visits* across exclusive diagnosis groups, for females and males separately over the study period 2018–2022.
**Table S29:** Birth year and birth year squared adjusted period prevalence and 95% confidence intervals, of *overall, in‐person, and digital SRH visits* across exclusive diagnosis groups, for females and males separately over the study period 2018–2022.
**Table S30:** Fully adjusted* period prevalence and 95% confidence intervals of *overall, in‐person, and digital SRH visits* across exclusive diagnosis groups, for females and males separately over the study period 2018–2022.
**Table S31:** Adjusted period prevalence and Odds Ratios of *SRH visits with midwives* at the population level across exclusive diagnosis groups over the study period 2018–2022.
**Table S32:** Adjusted period prevalence and Odds Ratios of *SRH visits with physicians* at the population level across exclusive diagnosis groups over the study period 2018–2022.
**Table S33:** Adjusted period prevalence and Odds Ratios of females aged 15–22 having used *contraceptives* (any, LARC, SARC) or *abortion* over the study period 2018–2022.
**Table S34:** Visits per 1000 individuals and Incident‐Rate Ratios (IRR) *of overall SRH visits* among youth with a youth clinic visit, stratified by sex and across exclusive diagnosis groups, over the study period.
**Table S35:** Visits per 1000 individuals and Incident‐Rate Ratios (IRR) *of in‐person SRH visits* among youth with a youth clinic visit, stratified by sex and across exclusive diagnosis groups, over the study period.
**Table S36:** Visits per 1000 individuals and Incident‐Rate Ratios (IRR) *of digital SRH visits* among youth with a youth clinic visit, stratified by sex and across exclusive diagnosis groups, over the study period.
**Table S37:** *Sociodemographic characteristics* of the study population by sex and by overlapping autism and ADHD groups among youth aged 12–22 in Stockholm County, 2018–2022.
**Table S38:** Sociodemographic characteristics of *SRH service users* by sex and by overlapping autism and ADHD groups among youth aged 12–22 in Stockholm County, 2018–2022.
**Table S39:** Adjusted period prevalence and Odds Ratios of *overall SRH visits* at the population level by overlapping autism and ADHD groups over the entire study period 2018–2022. Error bars represent 95% confidence interval.
**Table S42:** Adjusted period prevalence and Odds Ratios of females aged 15–22 having used *contraceptives* (any, LARC, SARC) or *abortion* over the study period 2018–2022 by overlapping autism and ADHD groups (*N* = 174,870).
**Table S43:** Adjusted period prevalence and Odds Ratios of visit with midwives by sex and overlapping autism and ADHD groups over the entire study period, 2018–2022.
**Table S44:** Adjusted period prevalence and Odds Ratios of visit with physicians by sex and overlapping autism and ADHD groups over the entire study period, 2018–2022.
**Table S45:** Visits per 1000 individuals and Incident‐Rate Ratios (IRR) of *overall SRH visits* by sex and by overlapping autism and ADHD groups over the study period (95% CI).
**Table S46:** Visits per 1000 individuals and Incident‐Rate Ratios (IRR) of *in‐person SRH visits* by sex and by overlapping autism and ADHD groups over the study period (95% CI).

## Data Availability

Swedish privacy law prohibits us from making registry data publicly available. The data supporting our findings was used under license and ethical approval for the current study. Readers interested in obtaining microdata or replicating our study may seek similar approvals and inquire through Statistics Sweden and Region Stockholm.
