# Supplementary material for: Sexual and Reproductive Healthcare Among Youth With Autism and ADHD: A Population‐Based Study From Sweden
Source: Autism Res. 2026 Jul 28;19(9):e70306. doi: 10.1002/aur.70306 (PMC13599709; doi:10.1002/aur.70306)
Supplement: Supplementary file 1 [file AUR-19-0-s001.docx]

# Supplements

**Supplementary Table 1. STROBE Statement—Checklist of items that should be included in reports of cohort studies**

|  | Item No | Recommendation |
| --- | --- | --- |
| **Title and abstract** | 1 | Indicate the study’s design with a commonly used term in the title or the abstract  *Sexual and Reproductive Healthcare Among Youth With Autism and ADHD: A Population-Based Study from Sweden* |
|  |  | (*b*) Provide in the abstract an informative and balanced summary of what was done and what was found  *Abstract, p. 3.* |
| Introduction | | |
| Background/rationale | 2 | Explain the scientific background and rationale for the investigation being reported  *Introduction, p.4-5.* |
| Objectives | 3 | State specific objectives, including any prespecified hypotheses  Introduction, p. 4-5. |
| Methods | | |
| Study design | 4 | Present key elements of study design early in the paper  *Methods, p. 5.* |
| Setting | 5 | Describe the setting, locations, and relevant dates, including periods of recruitment, exposure, follow-up, and data collection.  *Methods, p. 5-6, and Supplementary Figure 2 (flow chart of included contacts).* |
| Participants | 6 | (*a*) Give the eligibility criteria, and the sources and methods of selection of participants. Describe methods of follow-up.  *Study population section, p. 5 and supplementary Figure 1 in supplements and methods,* |
|  |  | (*b*) For matched studies, give matching criteria and number of exposed and unexposed  *N/A* |
| Variables | 7 | Clearly define all outcomes, exposures, predictors, potential confounders, and effect modifiers. Give diagnostic criteria, if applicable.  *Methods p. 6, section ‘Autism and ADHD’ on mutually exclusive diagnosis groups.*  *Also see Supplementary Table 2 for a detailed description of diagnostic groups.* |
| Data sources/ measurement | 8* | For each variable of interest, give sources of data and details of methods of assessment (measurement). Describe comparability of assessment methods if there is more than one group.  *See Supplementary Table 2 for a detailed description of variables, their sources, and coding.* |
| Bias | 9 | Describe any efforts to address potential sources of bias.  Methods: Primary analyses repeated with overlapping diagnostic groups, birth year as linear and square term, and data analyzed using only complete cases. |
| Study size | 10 | Explain how the study size was arrived at.  *Methods, study population, and Supplementary Figure 1 (flow chart of birth cohorts/study population).* |
| Quantitative variables | 11 | Explain how quantitative variables were handled in the analyses. If applicable, describe which groupings were chosen and why.  *Detailed description of birth year quintiles in Supplementary Table 2.* |
| Statistical methods | 12 | (*a*) Describe all statistical methods, including those used to control for confounding  *Methods section, statistical analysis p. 7-8.* |
|  |  | (*b*) Describe any methods used to examine subgroups and interactions  *Methods section, statistical analysis p. 7-8.* |
|  |  | (*c*) Explain how missing data were addressed  *Methods section, statistical analysis p. 7-8.* |
|  |  | (*d*) If applicable, explain how loss to follow-up was addressed  N/A |
|  |  | (*e*) Describe any sensitivity analyses  *Methods section, statistical analysis p. 7-8.* |
| Results | | |
| Participants | 13* | (a) Report numbers of individuals at each stage of study—eg numbers potentially eligible, examined for eligibility, confirmed eligible, included in the study, completing follow-up, and analyzed  *Supplementary Figure 1 - flowchart of individuals in the study population and annual populations.* |
|  |  | (b) Give reasons for non-participation at each stage  *N/A: No exclusions were made at population level.* |
|  |  | (c) Consider use of a flow diagram  *Supplementary Figure 1 - flowchart of individuals in the study population and annual populations.* |
| Descriptive data | 14* | (a) Give characteristics of study participants (eg demographic, clinical, social) and information on exposures and potential confounders  *Table 1, p. 17.* |
|  |  | (b) Indicate number of participants with missing data for each variable of interest  *Table 1, p. 17.* |
|  |  | (c) Summarise follow-up time (eg, average and total amount)  N/A |
| Outcome data | 15* | Report numbers of outcome events or summary measures over time  Supplementary Table 3 and Supplementary Table 17. |
| Main results | 16 | (*a*) Give unadjusted estimates and, if applicable, confounder-adjusted estimates and their precision (eg, 95% confidence interval). Make clear which confounders were adjusted for and why they were included.  *All unadjusted and adjusted estimates available in supplementary tables.* |
|  |  | (*b*) Report category boundaries when continuous variables were categorized  *Detailed description of birth year quintiles in Supplementary Table 2.*  1996-1999  2000-2002  2003-2005  2006-2008  2009-2010 |
|  |  | (*c*) If relevant, consider translating estimates of relative risk into absolute risk for a meaningful time period  *Tables with Odds Ratios also supplemented with period prevalence.* |
| Other analyses | 17 | Report other analyses done — e.g. analyses of subgroups and interactions, and sensitivity analyses  *Analyses using overlapping diagnostic groups, birth year as linear and square term, and complete cases are presented in supplement.* |
| Discussion | | |
| Key results | 18 | Summarise key results with reference to study objectives.  *First section in discussion, p. 11.* |
| Limitations | 19 | Discuss limitations of the study, taking into account sources of potential bias or imprecision. Discuss both direction and magnitude of any potential bias  *Strengths and weaknesses in discussion, p. 13-14.* |
| Interpretation | 20 | Give a cautious overall interpretation of results considering objectives, limitations, multiplicity of analyses, results from similar studies, and other relevant evidence.  *Discussion.* |
| Generalisability | 21 | Discuss the generalisability (external validity) of the study results.  *Strengths and limitations in discussion, p. 13-14.* |
| Other information | | |
| Funding | 22 | Give the source of funding and the role of the funders for the present study and, if applicable, for the original study on which the present article is based.  *Funding statement, p. 1.* |

**Supplementary Figure 1. Annual populations and study population, Stockholm residents 2018-2022.**

**Supplementary Table 2. Variable description and sources.**

| **Variable** | | **Comment** | **Source** |
| --- | --- | --- | --- |
| ***ID*** | *A project ID is used to identify unique individuals in the dataset. These are derived from a pseudonymized version of the personal identification number (PIN) of all individuals registered in the Stockholm region*^1^ | *Statistics Sweden* | |
| **Mutually exclusive diagnosis groups**  Control peers  Autism  ADHD  Autism & ADHD  **Overlapping diagnosis groups (sensitivity analysis)**  Control peers  Autism  ADHD | | Individuals with autism and ADHD were primarily identified using the International Classification of Diseases, 10th Revision (ICD-10)^2^, from regional administrative healthcare records covering specialist, in-patient, outpatient, and primary care (ICD-10: F84 & F90, respectively).  Autism spectrum diagnosis (ASD) was traced five years prior to each study year in regional administrative healthcare records (VAL – ORV/SLV). If identified within that window, individuals were classified as having autism/ASD for all study years, using a binary indicator variable.  For ADHD, diagnosis and prescriptions were traced five years prior to each study year. If identified within that window, individuals were classified as having ADHD for all study years, using a binary indicator variable.  Prescription records of dispensed centrally acting sympathomimetics (Anatomical Therapeutic Chemical (ATC) code N06BA) for ADHD treatment were obtained from regional administrative healthcare records (VAL-LAK). This register includes all dispensed medications included in the pharmaceutical benefits scheme, a list decided by The Dental and Pharmaceutical Benefits Agency.^3^ | **ICD-10 (diagnosis):** Regional administrative healthcare register (Vårdanalysdatabasen, VAL - OVR/SLV)  **ATC-codes (medical prescriptions):** Administrative healthcare register (Vårdanalysdatabasen, VAL-LAK) |
| **Sex**  Females  Males | | Individuals that have changed legal sex are not traceable, as their personal identification number is also changed in national registries. | Statistics Sweden^4-6^ |
| **Birth Year Quintiles**  1996-1999  2000-2002  2003-2005  2006-2008  2009-2010 | | Individuals were grouped into quintiles based on birth year. | Statistics Sweden^4-6^ |
| **Migrant background**  Swedish-born parents  Foreign-born parents  Foreign-born | | Derived from classifications of migrant background used by Swedish authorities like [Statistics Sweden](https://www.statistikdatabasen.scb.se/pxweb/sv/ssd/START__BE__BE0101__BE0101Q/UtlSvBakgFin/table/tableViewLayout1/?loadedQueryId=113580&timeType=from&timeValue=2020).   - Group (1) includes youth born in Sweden with either: two Swedish-born parents, one Swedish- and one foreign-born parent, or one identified parent identified born in Sweden. - Group (2) includes youth born in Sweden, with either: two foreign-born parents, or one identified parent born outside of Sweden. - Group (3) includes all youth born outside of Sweden. Aside from youth with own migration experience, this includes adoptees and youth born outside of Sweden with Swedish-born parents. | Statistics Sweden^4-6^ |
| **Maternal/paternal education**  Primary education  Upper secondary education  Post-secondary education | | Based classifications of educational degree available 2018-2020.  *Primary education*   - Pre-primary education - Primary and lower secondary education, (<9 years) - Primary and lower secondary education (>=9 years) - Upper secondary education (<2 years)   *Upper secondary education*   - Upper secondary education (>=2 years) - University education (<2 years)   *Post-secondary education*   - Higher education, (>=2 years) - Postgraduate/doctoral education | Statistics Sweden^4-6^ |
| **Household income** (quintiles, 1-5) | | Weighted income of parents adjusted for the birth year of offspring. The year with the maximum income level was used. Information available 2018-2020. | Statistics Sweden^4-6^ |
| ***Outcome variables*** | **Types of visits at youth clinics included:**  In-person visits  Video consultations  Chat consultations  **Appointments with medical staff at youth clinics:**  Midwives (and nurses)  Physicians  **Content of care (not restricted to youth clinics):**  Contraceptive dispensation  Abortion | - Regional Health Care Data Warehouse (VAL)*^7^ - TakeCare (electronic health record system)^8^ - Contraceptive dispensation (VAL-LAK)* - Abortion (VAL)*   The vast majority of visits at youth clinics are appointments with midwives, and on recommendation (more complex medical issues, underlying conditions or contraindications) physicians.  However, a small number of nurses work in a similar capacity as midwives – generally catering to male patients – why these midwives and nurses are coded as one staff category. In this analytical sample, 138 appointments were conducted with nurses compared to 436 333 midwife appointments.  **See description of VAL and VAL-LAK in the first row of this table, under “Mutually exclusive diagnosis groups”.* | |

1. Ludvigsson JF, Otterblad-Olausson P, Pettersson BU, Ekbom A. The Swedish personal identity number: possibilities and pitfalls in healthcare and medical research. Eur J Epidemiol. 2009 Nov 6;24(11):659–67.
2. Hirsch JA, Nicola G, McGinty G, Liu RW, Barr RM, Chittle MD, Manchikanti L. ICD-10: history and context. American Journal of Neuroradiology. 2016 Apr 1;37(4):596-9.
3. The Dental and Pharmaceutical Benefits Agency, TLV. Reimbursed medicines. [Internet]. 1 August 2022 [cited 2025 10 22]. Available from: [https://www.tlv.se/in-english/medicines/pricing-and-reimbursement-of-medicines/reimbursed-medicines.html](https://www.tlv.se/in-english/medicines/pricing-and-reimbursement-of-medicines/reimbursed-medicines.html?utm_source=chatgpt.com)
4. Ludvigsson JF, Almqvist C, Bonamy AKE, Ljung R, Michaëlsson K, Neovius M, et al. Registers of the Swedish total population and their use in medical research. Eur J Epidemiol. 2016 Feb 14;31(2):125–36.
5. Ekbom A. The Swedish Multi-generation Register. In 2011. p. 215–20.
6. Ludvigsson JF, Svedberg P, Olén O, Bruze G, Neovius M. The longitudinal integrated database for health insurance and labour market studies (LISA) and its use in medical research. Eur J Epidemiol. 2019 Apr 30;34(4):423–37.
7. Rajan G, Syding S, Ljunggren G, Wändell P, Wahlström L, Philips B, et al. Health care consumption and psychiatric diagnoses among adolescent girls 1 and 2 years after a first-time registered child sexual abuse experience: a cohort study in the Stockholm Region. Eur Child Adolesc Psychiatry. 2021 Nov 1;30(11):1803–11.
8. Cars T, Wettermark B, Malmström RE, Ekeving G, Vikström B, Bergman U, et al. Extraction of Electronic Health Record Data in a Hospital Setting: Comparison of Automatic and Semi‐Automatic Methods Using Anti‐Therapy as Model. Basic Clin Pharmacol Toxicol. 2013 Jun 7;112(6):392–400.

**Supplementary Figure 2. Flow-chart of included contacts at youth clinics in Stockholm, 2018-2022.**

**Supplementary Table 3. Trends in youth clinic *visits by year* and exclusive diagnosis groups, 2018-2022 (K=492 762).**

| Diagnosis group | 2018 | 2019 | 2020 | 2021 | 2022 | Total |
| --- | --- | --- | --- | --- | --- | --- |
| Total | 104 023 (21.1%) | 103 363 (21.0%) | 91 922 (18.7%) | 90 233 (18.3%) | 103 221 (20.9%) | 492 762 (100.0%) |
| Control peers | 88 999 (85.6%) | 87 885 (85.0%) | 77 303 (84.1%) | 75 816 (84.0%) | 86 779 (84.1%) | 416 782 (84.6%) |
| Autism | 1 861 (1.8%) | 1 875 (1.8%) | 1 857 (2.0%) | 1 971 (2.2%) | 2 345 (2.3%) | 9 909 (2.0%) |
| ADHD | 1 1186 (10.8%) | 11 479 (11.1%) | 10 789 (11.7%) | 10 440 (11.6%) | 11 712 (11.3%) | 55 606 (11.3%) |
| Autism & ADHD | 1 977 (1.9%) | 2 124 (2.1%) | 1 973 (2.1%) | 2 006 (2.2%) | 2 385 (2.3%) | 10 465 (2.1%) |

**Supplementary Table 4. Visits at Stockholm Youth Clinics by Diagnosis Group, Mode of Contact, and Healthcare Provider over the entire study period (K=492 762)*.***

| All contacts | Control peers | Autism | ADHD | Autism & ADHD | Total |
| --- | --- | --- | --- | --- | --- |
| Any SRH visit | 416 782 (100.0%) | 9 909 (100.0%) | 55 606 (100.0%) | 10 465 (100.0%) | 492 762 (100.0%) |
| In-person visit | 389 764 (93.5%) | 9 213 (93.0%) | 52 684 (94.7%) | 9 830 (93.9%) | 461 491 (93.7%) |
| Digital visit | 27 018 (6.5%) | 6 96 (7.0%) | 2 922 (5.3%) | 635 (6.1%) | 31 271 (6.3%) |
| Video | 18 586 (4.5%) | 433 (4.4%) | 1 956 (3.5%) | 450 (4.3%) | 21 425 (4.3%) |
| Chat | 8 432 (2.0%) | 263 (2.7%) | 966 (1.7%) | 185 (1.8%) | 9 846 (2.0%) |
| Midwife visit | 369 727 (88.7%) | 8 210 (82.9%) | 49 516 (89.0%) | 9 018 (86.2%) | 436 471 (88.6%) |
| Physician visit | 38 623 (9.3%) | 1 436 (14.5%) | 5 124 (9.2%) | 1 262 (12.1%) | 46 445 (9.4%) |
| *Missing healthcare provider information** | 8 432 (2.0%) | 263 (2.7%) | 966 (1.7%) | 185 (1.8%) | 9 846 (2.0%) |
|  | | | | | |
| Females | **Control peers** | **Autism** | **ADHD** | **Autism & ADHD** | **Total** |
| Any SRH visit | 375 200 (100.0%) | 9 267 (100.0%) | 50 051 (100.0%) | 9 577 (100.0%) | 444 095 (100.0%) |
| In-person visit | 349 631 (93.2%) | 8 612 (92.9%) | 47 296 (94.5%) | 8 979 (93.8%) | 414 518 (93.3%) |
| Digital visit | 25 569 (6.8%) | 655 (7.1%) | 2 755 (5.5%) | 598 (6.2%) | 29 577 (6.7%) |
| Video | 18 221 (4.9%) | 429 (4.6%) | 1896 (3.8%) | 442 (4.6%) | 20 988 (4.7%) |
| Chat | 7 348 (2.0%) | 226 (2.4%) | 859 (1.7%) | 156 (1.6%) | 8 589 (1.9%) |
| Midwife visit | 334 496 (89.2%) | 7 727 (83.4%) | 44 711 (89.3%) | 8 286 (86.5%) | 395 220 (89.0%) |
| Physician visit | 33 356(8.9%) | 1 314 (14.2%) | 4 481 (9.0%) | 1 135 (11.9%) | 40 286 (9.1%) |
| *Missing healthcare provider information** | 7 348 (2.0%) | 226 (2.4%) | 859 (1.7%) | 156 (1.6%) | 8 589 (1.9%) |
|  | | | | | |
| Males | **Control peers** | **Autism** | **ADHD** | **Autism & ADHD** | **Total** |
| Any SRH visit | 41 582 (100.0%) | 642 (100.0%) | 5 555 (100.0%) | 888 (100.0%) | 48 667 (100.0%) |
| In-person visit | 40 133 (96.5%) | 601 (93.6%) | 5 388 (97.0%) | 851 (95.8%) | 46 973 (96.5%) |
| Digital visit | 1 449 (3.5%) | 41 (6.4%) | 167 (3.0%) | 37 (4.2%) | 1 694 (3.5%) |
| Video | 365 (0.9%) | <10 (0.6%) | 60 (1.1%) | <10 (0.9%) | 437 (0.9%) |
| Chat | 1 084 (2.6%) | 37 (5.8%) | 107 (1.9%) | 29 (3.3%) | 1 257 (2.6%) |
| Midwife visit | 35 231 (84.7%) | 483 (75.2%) | 4 805 (86.5%) | 732 (82.4%) | 41 251 (84.8%) |
| Physician visit | 5 267 (12.7%) | 122 (19.0%) | 643 (11.6%) | 127 (14.3%) | 6 159 (12.7%) |
| *Missing healthcare provider information** | 1 084 (2.6%) | 37 (5.8%) | 107 (1.9%) | 29 (3.3%) | 1 257 (2.6%) |

***Chat contacts lacking information on healthcare provider.**

**Supplementary Table 5. Proportion among females ages 15-22 with any contraceptive prescription, long-acting reversible contraception, short-acting reversible contraception, or abortion over the study period 2018–2022 (N=174 870).**

|  | Any contraceptive | LARC | SARC | Abortion |
| --- | --- | --- | --- | --- |
| Control peers | 66 617 (54.9%) | 15 473 (12.8%) | 57 344 (47.3%) | 4 547 (3.7%) |
| Autism | 1 441 (52.0%) | 407 (14.7%) | 1 195 (43.2%) | 88 (3.2%) |
| ADHD | 6 489 (74.3%) | 2 136 (24.5%) | 5 329 (61.0%) | 810 (9.3%) |
| Autism & ADHD | 1 391 (66.1%) | 456 (21.7%) | 1 124 (53.4%) | 121 (5.8%) |
| Total | 75 938 (56.3%) | 18 472 (13.7%) | 64 992 (48.2%) | 5 566 (4.1%) |

**Supplementary Table 6. Sociodemographic characteristics of *SRH service use* by sex and exclusive diagnosis groups among youth aged 12–22 in Stockholm County, 2018–2022.**

|  | Females (N= 220 729) | | | | Males (N=233 676) | | | |
| --- | --- | --- | --- | --- | --- | --- | --- | --- |
|  | **87 121 (39.5%)** | | | | **21 774 (9.3%)** | | | |
| SRH service users | **Control peers** (n=99 228) | **Autism** (n=4 899) | **ADHD** (n=13 249) | **Autism & ADHD** (n=3 353) | **Control peers** (n**=**203 057) | **Autism** (n=6 951) | **ADHD** (n=17 895) | **Autism & ADHD** (n=5 773) |
| Proportion (%) with ≥1 SRH visit 2018-2022 | 75 592 (37.9%) | 1 805 (36.8%) | 7 975 (60.2%) | 1 749 (52.2%) | 18719.0 (9.2%) | 301 (4.3%) | 2 371(13.2%) | 383 (6.6%) |
| Mean visits over study period (SD) | 1.9 (3.6) | 1.9 (3.8) | 3.8 (5.2) | 2.9 (4.3) | 2.2 (2.0) | 2.1 (1.8) | 2.3 (2.1) | 2.3 (2.7) |
| Migrant background |  |  |  |  |  |  |  |  |
| Swedish-born parents | 57 236 (75.7%) | 1 515 (83.9%) | 6 609 (82.9%) | 1 541 (88.1%) | 12 120 (64.7%) | 235 (78.1%) | 1 803 (76.0%) | 316 (82.5%) |
| Foreign-born parents | 8 499 (11.2%) | 145 (8.0%) | 847 (10.6%) | 106 (6.1%) | 2 905 (15.5%) | 42 (14.0%) | 358 (15.1%) | 39 (10.2%) |
| Foreign-born | 9 857 (13.0%) | 145 (8.0%) | 519 (6.5%) | 102 (5.8%) | 3 694 (19.7%) | 24 (8.0% | 210 (8.9%) | 28 (7.3%) |
| Maternal education level |  |  |  |  |  |  |  |  |
| Primary education | 18 138 (24.0%) | 428 (23.7%) | 2 370 (29.7%) | 475 (27.2%) | 4 985 (26.6%) | 86 (28.6%) | 836 (35.3%) | 113 (29.5%) |
| Secondary education | 26 802 (35.5%) | 676 (37.5%) | 3 091 (38.8%) | 684 (39.1%) | 6 068 (32.4%) | 98 (32.6%) | 826 (34.8%) | 141 (36.8%) |
| Post-secondary education | 28 262 (37.4%) | 680 (37.7%) | 2 444 (30.6%) | 581 (33.2%) | 6 290 (33.6%) | 114 (37.9%) | 681 (28.7%) | 124 (32.4%) |
| Missing | 2 390 (3.2%) | 21 (1.2%) | 70 (0.9%) | 9 (0.5%) | 1 376 (7.4%) | <10 (1.0%) | 28 (1.2%) | <10 (1.3%) |
| Paternal education level |  |  |  |  |  |  |  |  |
| Primary education | 24 137 (31.9%) | 607 (33.6%) | 3 482 (43.7%) | 729 (41.7%) | 6 144 (32.8%) | 106 (35.2%) | 1 097 (46.3%) | 173 (45.2%) |
| Secondary education | 24 996 (33.1%) | 648 (35.9%) | 2 600 (32.6%) | 592 (33.8%) | 5 607 (30.0%) | 89 (29.6%) | 731 (30.8%) | 126 (32.9%) |
| Post-secondary education | 21 163 (28.0%) | 472 (26.1%) | 1 562 (19.6%) | 366 (20.9%) | 4 902 (26.2%) | 85 (28.2%) | 415 (17.5%) | 65 (17.0%) |
| Missing | 5 296 (7.0%) | 78 (4.3%) | 331 (4.2%) | 62 (3.5%) | 2 066 (11.0%) | 21 (7.0%) | 128 (5.4%) | 19 (5.0%) |
| Household income |  |  |  |  |  |  |  |  |
| Q1 | 10 867 (14.4%) | 308 (17.1%) | 1 491 (18.7%) | 336 (19.2%) | 3 276 (17.5%) | 81 (26.9%) | 523 (22.1%) | 80 (20.9%) |
| Q2 | 13 734 (18.2%) | 451 (25.0%) | 1 954 (24.5%) | 440 (25.2%) | 3 374 (18.0%) | 75 (24.9%) | 576 (24.3%) | 106 (27.7%) |
| Q3 | 15 511 (20.5%) | 443 (24.5%) | 1 718 (21.5%) | 406 (23.2%) | 3 483 (18.6%) | 55 (18.3%) | 497 (21.0%) | 83 (21.7%) |
| Q4 | 16 602 (22.0%) | 325 (18.0%) | 1 462 (18.3%) | 316 (18.1%) | 3 587 (19.2%) | 52 (17.3%) | 388 (16.4%) | 61 (15.9%) |
| Q5 | 17 008 (22.5%) | 257 (14.2%) | 1 307 (16.4%) | 241 (13.8%) | 3 979 (21.3%) | 35 (11.6%) | 374 (15.8%) | 49 (12.8%) |
| Missing | 18 70 (2.5%) | 21 (1.2%) | 43 (0.5%) | <10 (0.6%) | 1 020 (5.4%) | <10 (1.0%) | 13 (0.5%) | <10 (1.0%) |

**Supplementary Table 7. Period prevalence and Odds Ratios of *overall SRH visits* at the population level across exclusive diagnosis groups over the study period 2018–2022.**

| Overall visits | Adjusted for birth year quintiles | | Fully adjusted* | |
| --- | --- | --- | --- | --- |
|  | **Period prevalence (95% CI)** | **OR (95% CI)** | **Period prevalence (95% CI)** | **OR (95% CI)** |
| Females |  |  |  |  |
| Control peers | 26.9 (26.6, 27.2) | 1.00 | 34.5 (34.2, 34.8) | 1.00 |
| Autism | 25.4 (24.4, 26.5) | 0.91 (0.86, 0.97) | 27.4 (26.3, 28.6) | 0.68 (0.63, 0.73) |
| ADHD | 44.1 (43.2, 44.9) | 2.56 (2.46, 2.66) | 48.5 (47.6, 49.4) | 2.02 (1.93, 2.11) |
| Autism & ADHD | 36.4 (35.0, 37.9) | 1.71 (1.58, 1.84) | 38.7 (37.2, 40.3) | 1.24 (1.15, 1.35) |
| Males |  |  |  |  |
| Control peers | 4.7 (4.5, 4.9) | 1.00 | 6.3 (6.1, 6.5) | 1.00 |
| Autism | 2.5 (2.2, 2.8) | 0.50 (0.45, 0.57) | 3.1 (2.7, 3.5) | 0.46 (0.41, 0.52) |
| ADHD | 7.4 (7.0, 7.8) | 1.66 (1.58, 1.74) | 8.8 (8.4, 9.2) | 1.46 (1.39, 1.54) |
| Autism & ADHD | 3.6 (3.2, 4.0) | 0.74 (0.66, 0.82) | 4.3 (3.9, 4.8) | 0.65 (0.58, 0.73) |

*Adjusted for birth year quintiles and sociodemographic predictors (migrant background, parental education, and household income).

**Supplementary Table 8. Adjusted period prevalence and Odds Ratios of *in-person SRH visits* at the population level across exclusive diagnosis groups over the study period 2018–2022.**

| In-person | Birth-year adjusted | | Fully-adjusted* | |
| --- | --- | --- | --- | --- |
|  | **Period prevalence (95% CI)** | **OR (95% CI)** | **Period prevalence (95% CI)** | **OR (95% CI)** |
| Females |  |  |  |  |
| Control peers | 26.4 (26.1, 26.7) | 1.00 | 33.9 (33.5, 34.2) | 1.00 |
| Autism | 24.8 (23.8, 25.8) | 0.91 (0.85, 0.97) | 26.7 (25.6, 27.9) | 0.67 (0.63, 0.72) |
| ADHD | 43.5 (42.6, 44.3) | 2.56 (2.46, 2.66) | 47.8 (46.9, 48.7) | 2.02 (1.93, 2.11) |
| Autism & ADHD | 35.7 (34.3, 37.2) | 1.70 (1.58, 1.83) | 38.0 (36.5, 39.6) | 1.24 (1.14, 1.34) |
| Males |  |  |  |  |
| Control peers | 4.5 (4.3, 4.7) | 1.00 | 6.0 (5.8, 6.2) | 1.00 |
| Autism | 2.3 (2.0, 2.6) | 0.49 (0.43, 0.55) | 2.9 (2.5, 3.2) | 0.45 (0.40, 0.51) |
| ADHD | 7.0 (6.6, 7.4) | 1.66 (1.58, 1.74) | 8.4 (8.0, 8.8) | 1.46 (1.39, 1.54) |
| Autism & ADHD | 3.4 (3.0, 3.7) | 0.73 (0.66, 0.82) | 4.1 (3.6, 4.5) | 0.65 (0.58, 0.73) |

*Adjusted for birth year quintiles and sociodemographic predictors (migrant background, parental education, and household income).

**Supplementary Table 9. Adjusted period prevalence and Odds Ratios of *digital SRH visits* at the population level across exclusive diagnosis groups over the study period 2018–2022.**

| Digital | Adjusted for birth year quintiles | | Fully adjusted* | |
| --- | --- | --- | --- | --- |
|  | **Period prevalence (95% CI)** | **OR (95% CI)** | **Period prevalence (95% CI)** | **OR (95% CI)** |
| Females |  |  |  |  |
| Control peers | 3.9 (3.8, 4.1) | 1.00 | 5.3 (5.1, 5.5) | 1.00 |
| Autism | 3.6 (3.3, 4.1) | 0.92 (0.82, 1.03) | 4.3 (3.8, 4.8) | 0.79 (0.71, 0.89) |
| ADHD | 5.4 (5.1, 5.7) | 1.41 (1.33, 1.50) | 6.3 (6.0, 6.7) | 1.21 (1.14, 1.29) |
| Autism & ADHD | 4.3 (3.9, 4.9) | 1.11 (0.99, 1.25) | 4.9 (4.3, 5.5) | 0.91 (0.80, 1.03) |
| Males |  |  |  |  |
| Control peers | 0.3 (0.3, 0.4) | 1.00 | 0.4 (0.4, 0.4) | 1.00 |
| Autism | 0.3 (0.2, 0.4) | 0.80 (0.55, 1.16) | 0.3 (0.2, 0.5) | 0.79 (0.54, 1.15) |
| ADHD | 0.4 (0.4, 0.5) | 1.34 (1.12, 1.60) | 0.5 (0.4, 0.6) | 1.26 (1.05, 1.52) |
| Autism & ADHD | 0.3 (0.2, 0.4) | 0.94 (0.66, 1.34) | 0.4 (0.2, 0.5) | 0.89 (0.62, 1.27) |

*Adjusted for birth year quintiles and sociodemographic predictors (migrant background, parental education, and household income).

**Supplementary Table 10. Adjusted period prevalence and Odds Ratios of *video SRH visits* at the population level across exclusive diagnosis groups over the study period 2018–2022.**

| Video | Adjusted for birth year quintiles | | Fully adjusted* | |
| --- | --- | --- | --- | --- |
|  | **Period prevalence (95% CI)** | **OR (95% CI)** | **Period prevalence (95% CI)** | **OR (95% CI)** |
| Females |  |  |  |  |
| Control peers | 3.0 (2.9, 3.2) | 1.00 | 4.0 (3.9, 4.2) | 1.00 |
| Autism | 2.6 (2.2, 2.9) | 0.84 (0.74, 0.95) | 2.9 (2.6, 3.3) | 0.72 (0.63, 0.82) |
| ADHD | 4.1 (3.8, 4.4) | 1.38 (1.29, 1.47) | 4.7 (4.4, 5.0) | 1.19 (1.11, 1.27) |
| Autism & ADHD | 3.4 (3.0, 3.9) | 1.15 (1.01, 1.31) | 3.8 (3.4, 4.4) | 0.95 (0.83, 1.09) |
| Males |  |  |  |  |
| Control peers | 0.1 (0.1, 0.1) | 1.00 | 0.1 (0.1, 0.1) | 1.00 |
| Autism | 0.0 (0.0, 0.1) | 0.41 (0.15, 1.10) | 0.0 (0.0, 0.1) | 0.30 (0.10, 0.94) |
| ADHD | 0.2 (0.1, 0.2) | 1.75 (1.29, 2.37) | 0.2 (0.1, 0.2) | 1.53 (1.11, 2.10) |
| Autism & ADHD | 0.1 (0.0, 0.2) | 0.87 (0.43, 1.76) | 0.1 (0.0, 0.2) | 0.71 (0.34, 1.51) |

*Adjusted for birth year quintiles and sociodemographic predictors (migrant background, parental education, and household income).

**Supplementary Table 11. Adjusted period prevalence and Odds Ratios of *chat SRH visits* at the population level across exclusive diagnosis groups over the study period 2018–2022.**

| Chat | Adjusted for birth year quintiles | | Fully adjusted* | |
| --- | --- | --- | --- | --- |
|  | **Period prevalence (95% CI)** | **OR (95% CI)** | **Period prevalence (95% CI)** | **OR (95% CI)** |
| Females |  |  |  |  |
| Control peers | 1.3 (1.2, 1.4) | 1.00 | 1.8 (1.7, 1.9) | 1.00 |
| Autism | 1.3 (1.1, 1.6) | 0.98 (0.82, 1.17) | 1.6 (1.4, 1.9) | 0.88 (0.74, 1.05) |
| ADHD | 1.9 (1.7, 2.1) | 1.45 (1.32, 1.59) | 2.3 (2.1, 2.5) | 1.27 (1.15, 1.39) |
| Autism & ADHD | 1.3 (1.1, 1.6) | 0.99 (0.81, 1.21) | 1.5 (1.2, 1.8) | 0.79 (0.64, 0.98) |
| Males |  |  |  |  |
| Control peers | 0.2 (0.2, 0.3) | 1.00 | 0.3 (0.3, 0.3) | 1.00 |
| Autism | 0.2 (0.2, 0.3) | 0.93 (0.63, 1.38) | 0.3 (0.2, 0.4) | 0.96 (0.65, 1.42) |
| ADHD | 0.3 (0.2, 0.4) | 1.14 (0.92, 1.42) | 0.3 (0.3, 0.4) | 1.11 (0.89, 1.39) |
| Autism & ADHD | 0.2 (0.1, 0.3) | 0.91 (0.61, 1.37) | 0.3 (0.2, 0.4) | 0.90 (0.60, 1.36) |

*Adjusted for birth year quintiles and sociodemographic predictors (migrant background, parental education, and household income).

**Supplementary Table 12. Adjusted Odds Ratios and cross-model Comparisons (χ² Tests) of in-person and digital SRH visits across exclusive diagnosis group and sex over the study period (2018–2022).**

|  | In-person visit* | Digital visit* | Difference across in-person and digital visits* |
| --- | --- | --- | --- |
| Females | **OR (95% CI)** | **OR (95% CI)** | **P-value** |
| Control peers | 1.00 | 1.00 | - |
| Autism | 0.67 (0.63,0.72) | 0.79 (0.71,0.89) | 0.007 |
| ADHD | 2.02 (1.93,2.11) | 1.21 (1.14,1.29) | <0.001 |
| Autism & ADHD | 1.24 (1.14,1.34) | 0.91 (0.80,1.03) | <0.001 |
|  |  |  |  |
| Males | **OR (95% CI)** | **OR (95% CI)** | **P-value** |
| Control peers | 1.00 | 1.00 | - |
| Autism | 0.45 (0.40,0.51) | 0.79 (0.54,1.15) | 0.004 |
| ADHD | 1.46 (1.39,1.54) | 1.26 (1.05,1.52) | 0.125 |
| Autism & ADHD | 0.65 (0.58,0.73) | 0.89 (0.62,1.27) | 0.087 |

*Adjusted for birth year quintiles and sociodemographic predictors (migrant background, parental education, and household income).

**Supplementary Table 13. Visits per 1000 individuals and Incident-Rate Ratios (IRR) *of in-person SRH visits* among youth with a youth clinic visit, stratified by sex and across exclusive diagnosis groups, over the study period (95% CI).**

| **In-person SRH visits** |  | **Adjusted for birth year quintiles** | |  | **Fully adjusted*** | |
| --- | --- | --- | --- | --- | --- | --- |
|  | **Crude visits among visitors, mean (SD)** | **Adjusted rate per 1000 individuals**  **(95% CI)** | **Incidence rate ratio (95% CI)** |  | **Adjusted rate per 1000 individuals**  **(95% CI)** | **Incidence rate ratio (95% CI)** |
| **Females** |  |  |  |  |  |  |
| Control peers | 1.8 (3.4) | 1682 (1672, 1692) | 1.00 |  | 1943 (1932, 1955) | 1.00 |
| Autism | 1.8 (3.6) | 1789 (1748, 1829) | 1.06 (1.04, 1.09) |  | 2012 (1966, 2059) | 1.04 (1.01, 1.06) |
| ADHD | 3.6 (4.9) | 2181 (2159, 2204) | 1.30 (1.28, 1.31) |  | 2454 (2428, 2481) | 1.26 (1.25, 1.28) |
| Autism & ADHD | 2.7 (4.0) | 1924 (1882, 1965) | 1.14 (1.12, 1.17) |  | 2132 (2084, 2179) | 1.10 (1.07, 1.12) |
| **Males** |  |  |  |  |  |  |
| Control peers | 0.2 (0.9) | 190 (187, 193) | 1.00 |  | 212 (208, 215) | 1.00 |
| Autism | 0.1 (0.6) | 130 (116, 144) | 0.68 (0.61, 0.76) |  | 139 (123, 154) | 0.66 (0.59, 0.73) |
| ADHD | 0.3 (1.1) | 230 (223, 238) | 1.21 (1.17, 1.25) |  | 254 (246, 263) | 1.20 (1.16, 1.24) |
| Autism & ADHD | 0.1 (0.9) | 200 (183, 216) | 1.05 (0.96, 1.14) |  | 215 (196, 234) | 1.01 (0.93, 1.11) |
| *Adjusted for birth year quintiles and sociodemographic predictors (migrant background, parental education, and household income). | | | | | | |

**Supplementary Table 14. Visits per 1000 individuals and Incident-Rate Ratios (IRR) of *digital SRH visits* among youth with a youth clinic visit, stratified by sex and across exclusive diagnosis groups, over the study period (95% CI).**

| **Digital SRH visits** |  | **Adjusted for birth year quintiles** | |  | **Fully adjusted*** | |
| --- | --- | --- | --- | --- | --- | --- |
|  | **Crude visits among visitors, mean (SD)** | **Adjusted rate per 1000 individuals**  **(95% CI)** | **Incidence rate ratio (95% CI)** |  | **Adjusted rate per 1000 individuals**  **(95% CI)** | **Incidence rate ratio (95% CI)** |
| **Females** |  |  |  |  |  |  |
| Control peers | 0.1 (0.6) | 125 (123, 127) | 1.00 |  | 148 (145, 150) | 1.00 |
| Autism | 0.1 (0.6) | 127 (115, 139) | 1.02 (0.92, 1.12) |  | 146 (132, 160) | 0.99 (0.90, 1.09) |
| ADHD | 0.2 (0.8) | 147 (141, 154) | 1.18 (1.12, 1.23) |  | 170 (162, 177) | 1.15 (1.10, 1.20) |
| Autism & ADHD | 0.2 (0.7) | 141 (128, 154) | 1.13 (1.03, 1.24) |  | 154 (138, 169) | 1.04 (0.94, 1.15) |
| **Males** |  |  |  |  |  |  |
| Control peers | 0.0 (0.1) | 7 (7, 8) | 1.00 |  | 8 (8, 9) | 1.00 |
| Autism | 0.0 (0.1) | 6 (4, 8) | 0.85 (0.59, 1.21) |  | 7 (4, 9) | 0.83 (0.57, 1.19) |
| ADHD | 0.0 (0.1) | 8 (6, 9) | 1.07 (0.89, 1.29) |  | 9 (7, 10) | 1.05 (0.87, 1.27) |
| Autism & ADHD | 0.0 (0.1) | 6 (3, 8) | 0.78 (0.54, 1.13) |  | 6 (4, 9) | 0.76 (0.52, 1.11) |
| *Adjusted for birth year quintiles and sociodemographic predictors (migrant background, parental education, and household income). | | | | | | |

**Supplementary Table 15. Visits per 1000 individuals and Incident-Rate Ratios (IRR) of *overall SRH visits* among youth with a youth clinic visit, stratified by sex and across exclusive diagnosis groups, over the study period (95% CI).**

| **Overall SRH visits** |  | **Adjusted for birth year quintiles** | |  | **Fully adjusted*** | |
| --- | --- | --- | --- | --- | --- | --- |
|  | **Crude visits among visitors, mean (SD)** | **Adjusted rate per 1000 individuals**  **(95% CI)** | **Incidence rate ratio (95% CI)** |  | **Adjusted rate per 1000 individuals**  **(95% CI)** | **Incidence rate ratio (95% CI)** |
| **Females** |  |  |  |  |  |  |
| Control peers | 1.9 (3.6) | 1807 (1796, 1817) | 1.00 |  | 2087 (2075, 2100) | 1.00 |
| Autism | 1.9 (3.8) | 1899 (1858, 1940) | 1.05 (1.03, 1.07) |  | 2140 (2092, 2188) | 1.03 (1.00, 1.05) |
| ADHD | 3.8 (5.2) | 2298 (2275, 2322) | 1.27 (1.26, 1.28) |  | 2588 (2562, 2615) | 1.24 (1.23, 1.25) |
| Autism & ADHD | 2.9 (4.3) | 2031 (1989, 2074) | 1.12 (1.10, 1.15) |  | 2251 (2202, 2299) | 1.08 (1.06, 1.10) |
| **Males** |  |  |  |  |  |  |
| Control peers | 2.2 (2.0) | 197 (194, 200) | 1.00 |  | 219 (216, 223) | 1.00 |
| Autism | 2.1 (1.8) | 138 (124, 152) | 0.70 (0.63, 0.78) |  | 149 (133, 165) | 0.68 (0.61, 0.76) |
| ADHD | 2.3 (2.1) | 235 (228, 242) | 1.19 (1.16, 1.23) |  | 260 (251, 268) | 1.18 (1.15, 1.22) |
| Autism & ADHD | 2.3 (2.7) | 205 (188, 222) | 1.04 (0.96, 1.13) |  | 223 (203, 242) | 1.02 (0.93, 1.11) |
| *Adjusted for birth year quintiles and sociodemographic predictors (migrant background, parental education, and household income). | | | | | | |

**Supplementary Table 16. Adjusted period prevalence and Odds Ratios of *SRH visits with midwives* at the population level across exclusive diagnosis groups over the study period 2018–2022.**

| Midwives | Adjusted for birth year quintiles | | Fully adjusted* | |
| --- | --- | --- | --- | --- |
|  | **Period prevalence (95% CI)** | **OR (95% CI)** | **Period prevalence (95% CI)** | **OR (95% CI)** |
| Females |  |  |  |  |
| Control peers | 25.4 (25.1, 25.7) | 1.00 | 32.9 (32.6, 33.2) | 1.00 |
| Autism | 23.1 (22.2, 24.2) | 0.86 (0.81, 0.92) | 25.1 (24.0, 26.3) | 0.64 (0.60, 0.69) |
| ADHD | 42.0 (41.2, 42.9) | 2.56 (2.46, 2.67) | 46.7 (45.8, 47.6) | 2.02 (1.94, 2.11) |
| Autism & ADHD | 34.0 (32.6, 35.4) | 1.66 (1.54, 1.79) | 36.4 (34.9, 38.0) | 1.21 (1.11, 1.31) |
| Males |  |  |  |  |
| Control peers | 4.1 (3.9, 4.3) | 1.00 | 5.5 (5.3, 5.7) | 1.00 |
| Autism | 2.0 (1.8, 2.3) | 0.48 (0.42, 0.54) | 2.5 (2.2, 2.9) | 0.44 (0.38, 0.50) |
| ADHD | 6.5 (6.1, 6.9) | 1.69 (1.61, 1.77) | 7.9 (7.5, 8.3) | 1.49 (1.41, 1.56) |
| Autism & ADHD | 3.0 (2.7, 3.4) | 0.71 (0.64, 0.80) | 3.6 (3.2, 4.0) | 0.63 (0.56, 0.71) |

*Adjusted for birth year quintiles and sociodemographic predictors (migrant background, parental education, and household income).

**Supplementary Table 17. Adjusted period prevalence and Odds Ratios of *SRH visits with physicians* at the population level across exclusive diagnosis groups over the study period 2018–2022.**

|  | Adjusted for birth year quintiles | | Fully adjusted* | |
| --- | --- | --- | --- | --- |
|  | **Period prevalence (95% CI)** | **OR (95% CI)** | **Period prevalence (95% CI)** | **OR (95% CI)** |
| Females |  |  |  |  |
| Control peers | 8.3 (8.2, 8.5) | 1.00 | 10.6 (10.4, 10.8) | 1.00 |
| Autism | 11.7 (11.0, 12.6) | 1.48 (1.36, 1.60) | 12.8 (11.9, 13.7) | 1.24 (1.14, 1.35) |
| ADHD | 15.0 (14.5, 15.6) | 1.99 (1.90, 2.08) | 16.4 (15.8, 17.1) | 1.68 (1.60, 1.76) |
| Autism & ADHD | 14.9 (13.8, 16.0) | 1.96 (1.80, 2.14) | 15.8 (14.7, 17.0) | 1.60 (1.46, 1.75) |
| Males |  |  |  |  |
| Control peers | 1.0 (0.9, 1.1) | 1.00 | 1.3 (1.2, 1.4) | 1.00 |
| Autism | 0.7 (0.5, 0.9) | 0.67 (0.54, 0.85) | 0.9 (0.7, 1.1) | 0.64 (0.51, 0.81) |
| ADHD | 1.4 (1.2, 1.5) | 1.38 (1.24, 1.52) | 1.6 (1.4, 1.8) | 1.23 (1.11, 1.37) |
| Autism & ADHD | 0.8 (0.6, 1.0) | 0.77 (0.62, 0.97) | 0.9 (0.7, 1.2) | 0.71 (0.56, 0.89) |

*Adjusted for birth year quintiles and sociodemographic predictors (migrant background, parental education, and household income).

**Supplementary Figure** **3. Unadjusted and adjusted odds ratios (OR) and 95% CIs of content of care among females aged 15-22 over the study period.** Content shows abortion services and prescriptions of long-acting reversible contraceptives (LARC) and short-acting reversible contraceptives (SARC) among autistic and/or ADHD youth compared to non-diagnosed control peers for the entire study period 2018-2022. Model 1 adjusted for birth year, Model 2 adjusted for birth year, migrant background, parental education, and household income. Error bars represent 95% CI.

**Supplementary Table 18. Adjusted period prevalence and Odds Ratios of females aged 15-22 having used *contraceptives* (any, LARC, SARC) or *abortion* over the study period 2018–2022 (N=174 870).**

|  | Adjusted for birth year quintiles | | Fully adjusted* | |
| --- | --- | --- | --- | --- |
|  | **Period prevalence (95% CI)** | **OR (95% CI)** | **Period prevalence (95% CI)** | **OR (95% CI)** |
| Any contraceptive |  |  |  |  |
| Control peers | 54.7 (54.5, 55.0) | 1.00 | 59.3 (59.0, 59.7) | 1.00 |
| Autism | 50.6 (48.8, 52.5) | 0.85 (0.79, 0.91) | 47.4 (45.6, 49.3) | 0.60 (0.55, 0.65) |
| ADHD | 73.0 (72.1, 74.0) | 2.25 (2.14, 2.36) | 70.6 (69.5, 71.6) | 1.69 (1.61, 1.79) |
| Autism & ADHD | 64.1 (62.1, 66.2) | 1.48 (1.35, 1.62) | 59.2 (57.1, 61.5) | 1.00 (0.90, 1.10) |
| LARC |  |  |  |  |
| Control peers | 12.6 (12.4, 12.8) | 1.00 | 13.3 (13.1, 13.6) | 1.00 |
| Autism | 14.3 (13.0, 15.6) | 1.16 (1.04, 1.29) | 13.5 (12.3, 14.8) | 1.01 (0.91, 1.13) |
| ADHD | 24.1 (23.2, 25.0) | 2.21 (2.10, 2.33) | 22.6 (21.7, 23.5) | 1.91 (1.81, 2.02) |
| Autism & ADHD | 21.1 (19.4, 22.9) | 1.87 (1.68, 2.07) | 19.3 (17.7, 21.0) | 1.56 (1.40, 1.74) |
| SARC |  |  |  |  |
| Control peers | 47.0 (46.7, 47.3) | 1.00 | 51.3 (51.0, 51.7) | 1.00 |
| Autism | 41.6 (39.8, 43.4) | 0.80 (0.74, 0.87) | 39.4 (37.6, 41.3) | 0.60 (0.55, 0.65) |
| ADHD | 59.1 (58.1, 60.2) | 1.64 (1.57, 1.72) | 56.7 (55.6, 57.8) | 1.26 (1.20, 1.32) |
| Autism & ADHD | 50.9 (48.8, 53.1) | 1.17 (1.08, 1.28) | 47.0 (44.9, 49.2) | 0.83 (0.76, 0.92) |
| Abortion |  |  |  |  |
| Control peers | 3.4 (3.3, 3.5) | 1.00 | 3.3 (3.1, 3.4) | 1.00 |
| Autism | 3.0 (2.4, 3.7) | 0.87 (0.70, 1.08) | 2.8 (2.2, 3.4) | 0.84 (0.67, 1.04) |
| ADHD | 9.1 (8.5, 9.7) | 2.83 (2.62, 3.06) | 7.5 (6.9, 8.0) | 2.41 (2.21, 2.62) |
| Autism & ADHD | 5.6 (4.7, 6.7) | 1.69 (1.40, 2.04) | 4.5 (3.8, 5.5) | 1.42 (1.16, 1.72) |

*Adjusted for birth year quintiles and sociodemographic predictors (migrant background, parental education, and household income).

## Complete cases

**Supplementary Table 19. Adjusted period prevalence, odds ratios, and 95% confidence intervals, of *overall, in-person, and digital SRH visits* across exclusive diagnosis groups, for females and males separately over the study period 2018–2022, using only complete cases (N=356 758).**

|  | **Overall visits** | | | |  | **In-person visits** | |  | | **Digital visits** | |
| --- | --- | --- | --- | --- | --- | --- | --- | --- | --- | --- | --- |
|  | **Period prevalence**  **(95% CI)** | | **Odds ratio**  **(95% CI)** | |  | **Period prevalence**  **(95% CI)** | **Odds ratio**  **(95% CI)** |  | | **Period prevalence**  **(95% CI)** | **Odds ratio**  **(95% CI)** |
| **Females** |  | |  | |  |  |  |  | |  |  |
| Control peers | 35.76 (35.43, 36.09) | | 1.00 | |  | 35.15 (34.82, 35.48) | 1.00 |  | | 5.64 (5.47, 5.82) | 1.00 |
| Autism | 30.47 (29.25, 31.75) | | 0.76 (0.71, 0.81) | |  | 29.75 (28.54, 31.02) | 0.75 (0.71, 0.81) |  | | 4.76 (4.27, 5.29) | 0.83 (0.74, 0.93) |
| ADHD | 52.16 (51.25, 53.08) | | 2.21 (2.12, 2.31) | |  | 51.53 (50.62, 52.45) | 2.22 (2.12, 2.31) |  | | 6.99 (6.60, 7.40) | 1.27 (1.19, 1.34) |
| Autism & ADHD | 43.06 (41.45, 44.73) | | 1.43 (1.32, 1.55) | |  | 42.37 (40.76, 44.03) | 1.43 (1.32, 1.54) |  | | 5.46 (4.87, 6.12) | 0.96 (0.85, 1.09) |
| **Males** | |  | |  | |  |  |  |  |  |  |
| Control peers | 6.34 (6.15, 6.54) | | 1.00 | |  | 6.06 (5.87, 6.26) | 1.00 |  | | 0.41 (0.37, 0.45) | 1.00 |
| Autism | 3.06 (2.71, 3.44) | | 0.45 (0.40, 0.51) | |  | 2.84 (2.51, 3.20) | 0.44 (0.39, 0.50) |  | | 0.32 (0.22, 0.46) | 0.77 (0.53, 1.13) |
| ADHD | 8.91 (8.49, 9.35) | | 1.47 (1.40, 1.55) | |  | 8.53 (8.12, 8.96) | 1.47 (1.40, 1.55) |  | | 0.51 (0.43, 0.62) | 1.26 (1.05, 1.52) |
| Autism & ADHD | 4.27 (3.84, 4.74) | | 0.65 (0.58, 0.72) | |  | 4.05 (3.64, 4.50) | 0.64 (0.57, 0.72) |  | | 0.36 (0.25, 0.51) | 0.88 (0.62, 1.26) |

*Adjusted for birth year quintiles.

**Supplementary Table 20. Fully adjusted* period prevalence, odds ratios, and 95% confidence intervals, of *overall, in-person, and digital SRH visits* across exclusive diagnosis groups, for females and males separately over the study period 2018–2022, using only complete cases (N=356 758).**

|  | **Overall visits** | | | |  | **In-person visits** | | |  | | **Digital visits** | |
| --- | --- | --- | --- | --- | --- | --- | --- | --- | --- | --- | --- | --- |
|  | **Period prevalence**  **(95% CI)** | | **Odds ratio**  **(95% CI)** | |  | **Period prevalence**  **(95% CI)** | **Odds ratio**  **(95% CI)** | |  | | **Period prevalence**  **(95% CI)** | **Odds ratio**  **(95% CI)** |
| **Females** |  | |  | |  |  |  | |  | |  |  |
| Control peers | 44.7 (44.5, 44.9) | | 1.00 | |  | 44.1 (43.9, 44.3) | 1.00 | |  | | 8.7 (8.6, 8.9) | 1.00 |
| Autism | 37.2 (35.9, 38.5) | | 0.68 (0.63, 0.73) | |  | 36.5 (35.2, 37.7) | 0.67 (0.63, 0.72) | |  | | 7.1 (6.4, 7.8) | 0.79 (0.71, 0.89) |
| ADHD | 58.1 (57.3, 58.9) | | 2.02 (1.93, 2.11) | |  | 57.5 (56.8, 58.3) | 2.02 (1.93, 2.11) | |  | | 10.3 (9.8, 10.8) | 1.21 (1.14, 1.29) |
| Autism & ADHD | 49.0 (47.4, 50.5) | | 1.24 (1.15, 1.35) | |  | 48.3 (46.7, 49.8) | 1.24 (1.14, 1.34) | |  | | 8.0 (7.2, 8.9) | 0.91 (0.80, 1.03) |
| **Males** | |  | |  | |  |  |  | |  |  |  |
| Control peers | 10.4 (10.3, 10.6) | | 1.00 | |  | 10.2 (10.1, 10.3) | 1.00 | |  | | 0.6 (0.6, 0.7) | 1.00 |
| Autism | 5.3 (4.7, 5.8) | | 0.46 (0.41, 0.52) | |  | 5.0 (4.4, 5.6) | 0.45 (0.40, 0.51) | |  | | 0.5 (0.3, 0.7) | 0.79 (0.54, 1.15) |
| ADHD | 14.2 (13.7, 14.7) | | 1.46 (1.39, 1.54) | |  | 13.9 (13.4, 14.5) | 1.46 (1.39, 1.54) | |  | | 0.8 (0.7, 0.9) | 1.26 (1.05, 1.52) |
| Autism & ADHD | 7.2 (6.5, 7.9) | | 0.65 (0.58, 0.73) | |  | 7.0 (6.3, 7.7) | 0.65 (0.58, 0.73) | |  | | 0.6 (0.4, 0.8) | 0.89 (0.62, 1.27) |
| *Adjusted for birth year quintiles and sociodemographic predictors (migrant background, parental education, and household income). | | | | | | | | | | | | |

**Supplementary Table 21. Adjusted period prevalence and Odds Ratios of *SRH visits with midwives* at the population level across exclusive diagnosis groups over the study period 2018–2022, using only complete cases (N=356 758).**

| Midwives | Adjusted for birth year quintiles |  | Fully adjusted* |  |
| --- | --- | --- | --- | --- |
|  | **Period prevalence (95% CI)** | **OR (95% CI)** | **Period prevalence (95% CI)** | **OR (95% CI)** |
| Females |  |  |  |  |
| Control peers | 34.2 (33.9, 34.6) | 1.00 | 32.9 (32.6, 33.2) | 1.00 |
| Autism | 28.1 (26.9, 29.3) | 0.72 (0.67, 0.77) | 25.1 (24.0, 26.3) | 0.64 (0.60, 0.69) |
| ADHD | 50.4 (49.5, 51.3) | 2.22 (2.13, 2.32) | 46.7 (45.8, 47.6) | 2.02 (1.94, 2.11) |
| Autism & ADHD | 40.8 (39.2, 42.4) | 1.39 (1.29, 1.51) | 36.4 (34.9, 38.0) | 1.21 (1.11, 1.31) |
| Males |  |  |  |  |
| Control peers | 5.6 (5.4, 5.8) | 1.00 | 5.5 (5.3, 5.7) | 1.00 |
| Autism | 2.5 (2.2, 2.9) | 0.43 (0.37, 0.49) | 2.5 (2.2, 2.9) | 0.44 (0.38, 0.50) |
| ADHD | 8.0 (7.6, 8.4) | 1.50 (1.42, 1.58) | 7.9 (7.5, 8.3) | 1.49 (1.41, 1.56) |
| Autism & ADHD | 3.6 (3.2, 4.0) | 0.62 (0.55, 0.70) | 3.6 (3.2, 4.0) | 0.63 (0.56, 0.71) |

*Adjusted for birth year quintiles and sociodemographic predictors (migrant background, parental education, and household income)

**Supplementary Table 22. Adjusted period prevalence and Odds Ratios of *SRH visits with physicians* at the population level across exclusive diagnosis groups over the study period 2018–2022, using only complete cases (N=356 758).**

| Physicians | Adjusted for birth year quintiles |  | Fully adjusted* |  |
| --- | --- | --- | --- | --- |
|  | **Period prevalence (95% CI)** | **OR (95% CI)** | **Period prevalence (95% CI)** | **OR (95% CI)** |
| Females |  |  |  |  |
| Control peers | 10.9 (10.7, 11.1) | 1.00 | 10.6 (10.4, 10.8) | 1.00 |
| Autism | 13.8 (12.9, 14.8) | 1.32 (1.21, 1.43) | 12.8 (11.9, 13.7) | 1.24 (1.14, 1.35) |
| ADHD | 17.7 (17.1, 18.4) | 1.79 (1.71, 1.87) | 16.4 (15.8, 17.1) | 1.68 (1.60, 1.76) |
| Autism & ADHD | 17.3 (16.1, 18.6) | 1.74 (1.59, 1.90) | 15.8 (14.7, 17.0) | 1.60 (1.46, 1.75) |
| Males |  |  |  |  |
| Control peers | 1.4 (1.3, 1.5) | 1.00 | 1.3 (1.2, 1.4) | 1.00 |
| Autism | 0.9 (0.7, 1.1) | 0.63 (0.50, 0.80) | 0.9 (0.7, 1.1) | 0.64 (0.51, 0.81) |
| ADHD | 1.7 (1.5, 1.9) | 1.23 (1.10, 1.36) | 1.6 (1.4, 1.8) | 1.23 (1.11, 1.37) |
| Autism & ADHD | 1.0 (0.8, 1.2) | 0.71 (0.57, 0.89) | 0.9 (0.7, 1.2) | 0.71 (0.56, 0.89) |

*Adjusted for birth year quintiles and sociodemographic predictors (migrant background, parental education, and household income)

**Supplementary Table 23. Adjusted period prevalence and Odds Ratios of females aged 15-22 having used *contraceptives* (any, LARC, SARC) or *abortion* over the study period 2018–2022, using only complete cases (N=110 216).**

|  | Adjusted for birth year quintiles | | Fully adjusted* | |
| --- | --- | --- | --- | --- |
|  | **Period prevalence (95% CI)** | **OR (95% CI)** | **Period prevalence (95% CI)** | **OR (95% CI)** |
| Any contraceptive |  |  |  |  |
| Control peers | 61.2 (60.9, 61.5) | 1.00 | 59.3 (59.0, 59.7) | 1.00 |
| Autism | 51.6 (49.7, 53.6) | 0.67 (0.62, 0.73) | 47.4 (45.6, 49.3) | 0.60 (0.55, 0.65) |
| ADHD | 74.1 (73.2, 75.1) | 1.82 (1.73, 1.92) | 70.6 (69.5, 71.6) | 1.69 (1.61, 1.79) |
| Autism & ADHD | 64.5 (62.4, 66.7) | 1.15 (1.05, 1.27) | 59.2 (57.1, 61.5) | 1.00 (0.90, 1.10) |
| LARC |  |  |  |  |
| Control peers | 13.6 (13.4, 13.8) | 1.00 | 13.3 (13.1, 13.6) | 1.00 |
| Autism | 14.5 (13.2, 15.9) | 1.08 (0.96, 1.20) | 13.5 (12.3, 14.8) | 1.01 (0.91, 1.13) |
| ADHD | 24.1 (23.2, 25.1) | 2.02 (1.92, 2.13) | 22.6 (21.7, 23.5) | 1.91 (1.81, 2.02) |
| Autism & ADHD | 21.1 (19.4, 22.9) | 1.70 (1.52, 1.89) | 19.3 (17.7, 21.0) | 1.56 (1.40, 1.74) |
| SARC |  |  |  |  |
| Control peers | 53.1 (52.8, 53.4) | 1.00 | 51.3 (51.0, 51.7) | 1.00 |
| Autism | 42.7 (40.8, 44.6) | 0.66 (0.61, 0.71) | 39.4 (37.6, 41.3) | 0.60 (0.55, 0.65) |
| ADHD | 60.4 (59.4, 61.5) | 1.35 (1.29, 1.42) | 56.7 (55.6, 57.8) | 1.26 (1.20, 1.32) |
| Autism & ADHD | 51.6 (49.5, 53.9) | 0.94 (0.86, 1.03) | 47.0 (44.9, 49.2) | 0.83 (0.76, 0.92) |
| Abortion |  |  |  |  |
| Control peers | 3.5 (3.3, 3.6) | 1.00 | 3.3 (3.1, 3.4) | 1.00 |
| Autism | 3.1 (2.5, 3.8) | 0.89 (0.72, 1.11) | 2.8 (2.2, 3.4) | 0.84 (0.67, 1.04) |
| ADHD | 8.7 (8.1, 9.4) | 2.68 (2.46, 2.91) | 7.5 (6.9, 8.0) | 2.41 (2.21, 2.62) |
| Autism & ADHD | 5.4 (4.5, 6.5) | 1.59 (1.31, 1.93) | 4.5 (3.8, 5.5) | 1.42 (1.16, 1.72) |

*Adjusted for birth year quintiles and sociodemographic predictors (migrant background, parental education, and household income).

**Supplementary Table 24. Visits per 1000 individuals and Incident-Rate Ratios (IRR) *of overall SRH visits* among youth with a youth clinic visit, stratified by sex and across exclusive diagnosis groups, over the study period using only complete cases (N=356 758)**

| **Overall SRH visits** |  | **Adjusted for birth year quintiles** | |  | **Fully adjusted*** | |
| --- | --- | --- | --- | --- | --- | --- |
|  | **Crude visits among visitors, mean (SD)** | **Adjusted rate per 1000 individuals**  **(95% CI)** | **Incidence rate ratio (95% CI)** |  | **Adjusted rate per 1000 individuals**  **(95% CI)** | **Incidence rate ratio (95% CI)** |
| **Females** |  |  |  |  |  |  |
| Control peers | 2.2 (3.8) | 2106 (2093, 2118) | 1.00 |  | 2087 (2075, 2100) | 1.00 |
| Autism | 2.0 (4.0) | 2185 (2136, 2233) | 1.04 (1.02, 1.06) |  | 2140 (2092, 2188) | 1.03 (1.00, 1.05) |
| ADHD | 4.0 (5.2) | 2650 (2623, 2678) | 1.26 (1.25, 1.27) |  | 2588 (2562, 2615) | 1.24 (1.23, 1.25) |
| Autism & ADHD | 2.9 (4.3) | 2312 (2263, 2362) | 1.10 (1.08, 1.12) |  | 2251 (2202, 2299) | 1.08 (1.06, 1.10) |
| **Males** |  |  |  |  |  |  |
| Control peers | 0.2 (0.9) | 219 (216, 222) | 1.00 |  | 219 (216, 223) | 1.00 |
| Autism | 0.1 (0.6) | 149 (133, 165) | 0.68 (0.61, 0.76) |  | 149 (133, 165) | 0.68 (0.61, 0.76) |
| ADHD | 0.3 (1.1) | 261 (252, 269) | 1.19 (1.15, 1.23) |  | 260 (251, 268) | 1.18 (1.15, 1.22) |
| Autism & ADHD | 0.2 (0.9) | 222 (202, 241) | 1.01 (0.93, 1.10) |  | 223 (203, 242) | 1.02 (0.93, 1.11) |
| *Adjusted for birth year quintiles and sociodemographic predictors (migrant background, parental education, and household income). | | | | | | |

**Supplementary Table 25. Visits per 1000 individuals and Incident-Rate Ratios (IRR) *of in-person SRH visits* among youth with a youth clinic visit, stratified by sex and across exclusive diagnosis groups, over the study period using only complete cases (N=356 758)**

| **In-person SRH visits** |  | **Adjusted for birth year quintiles** | |  | **Fully adjusted*** | |
| --- | --- | --- | --- | --- | --- | --- |
|  | **Crude visits among visitors, mean (SD)** | **Adjusted rate per 1000 individuals**  **(95% CI)** | **Incidence rate ratio (95% CI)** |  | **Adjusted rate per 1000 individuals**  **(95% CI)** | **Incidence rate ratio (95% CI)** |
| **Females** |  |  |  |  |  |  |
| Control peers | 2.1 (3.6) | 1956 (1944, 1968) | 1.00 |  | 1943 (1932, 1955) | 1.00 |
| Autism | 1.9 (3.7) | 2054 (2006, 2101) | 1.05 (1.03, 1.07) |  | 2012 (1966, 2059) | 1.04 (1.01, 1.06) |
| ADHD | 3.8 (5.0) | 2515 (2489, 2542) | 1.29 (1.27, 1.30) |  | 2454 (2428, 2481) | 1.26 (1.25, 1.28) |
| Autism & ADHD | 2.8 (4.0) | 2192 (2143, 2240) | 1.12 (1.10, 1.15) |  | 2132 (2084, 2179) | 1.10 (1.07, 1.12) |
| **Males** |  |  |  |  |  |  |
| Control peers | 0.2 (0.9) | 211 (208, 215) | 1.00 |  | 212 (208, 215) | 1.00 |
| Autism | 0.1 (0.6) | 139 (123, 154) | 0.66 (0.59, 0.73) |  | 139 (123, 154) | 0.66 (0.59, 0.73) |
| ADHD | 0.3 (1.1) | 255 (247, 264) | 1.21 (1.17, 1.25) |  | 254 (246, 263) | 1.20 (1.16, 1.24) |
| Autism & ADHD | 0.2 (0.9) | 214 (195, 233) | 1.01 (0.93, 1.11) |  | 215 (196, 234) | 1.01 (0.93, 1.11) |
| *Adjusted for birth year quintiles and sociodemographic predictors (migrant background, parental education, and household income). | | | | | | |

**Supplementary Table 26. Visits per 1000 individuals and Incident-Rate Ratios (IRR) *of digital SRH visits* among youth with a youth clinic visit, stratified by sex and across exclusive diagnosis groups, over the study period using only complete cases (N=356 758)**

| **Digital SRH visits** |  | **Adjusted for birth year quintiles** | |  | **Fully adjusted*** | |
| --- | --- | --- | --- | --- | --- | --- |
|  | **Crude visits among visitors, mean (SD)** | **Adjusted rate per 1000 individuals**  **(95% CI)** | **Incidence rate ratio (95% CI)** |  | **Adjusted rate per 1000 individuals**  **(95% CI)** | **Incidence rate ratio (95% CI)** |
| **Females** |  |  |  |  |  |  |
| Control peers | 0.2 (0.7) | 151 (148, 154) | 1.00 |  | 148 (145, 150) | 1.00 |
| Autism | 0.1 (0.7) | 147 (133, 161) | 0.97 (0.88, 1.07) |  | 146 (132, 160) | 0.99 (0.90, 1.09) |
| ADHD | 0.2 (0.8) | 170 (163, 178) | 1.13 (1.08, 1.18) |  | 170 (162, 177) | 1.15 (1.10, 1.20) |
| Autism & ADHD | 0.2 (0.7) | 156 (141, 172) | 1.03 (0.94, 1.14) |  | 154 (138, 169) | 1.04 (0.94, 1.15) |
| **Males** |  |  |  |  |  |  |
| Control peers | 0.0 (0.1) | 8 (8, 9) | 1.00 |  | 8 (8, 9) | 1.00 |
| Autism | 0.0 (0.1) | 7 (4, 9) | 0.83 (0.57, 1.19) |  | 7 (4, 9) | 0.83 (0.57, 1.19) |
| ADHD | 0.0 (0.1) | 9 (7, 10) | 1.04 (0.86, 1.26) |  | 9 (7, 10) | 1.05 (0.87, 1.27) |
| Autism & ADHD | 0.0 (0.1) | 6 (4, 9) | 0.75 (0.51, 1.10) |  | 6 (4, 9) | 0.76 (0.52, 1.11) |
| *Adjusted for birth year quintiles and sociodemographic predictors (migrant background, parental education, and household income). | | | | | | |

## Birth year and birth year squared-adjusted models

**Supplementary Table 27. Birth year and birth year squared adjusted odds ratios, and 95% confidence intervals, of *overall, in-person, and digital SRH visits* across exclusive diagnosis groups, for females and males separately over the study period 2018–2022.**

|  | **Overall visits** | **In-person visits** | **Digital visits** | **Video** | | **chat** |
| --- | --- | --- | --- | --- | --- | --- |
|  | **Odds ratio**  **(95% CI)** | **Odds ratio**  **(95% CI)** | **Odds ratio**  **(95% CI)** | **Odds ratio**  **(95% CI)** | | **Odds ratio**  **(95% CI)** |
| **Females** |  |  |  |  | |  |
| Control peers | 1.00 | 1.00 | 1.00 | 1.00 | | 1.00 |
| Autism | 0.90 (0.84, 0.96) | 0.89 (0.84, 0.95) | 0.90 (0.80, 1.00) | 0.82 (0.72, 0.93) | | 0.96 (0.80, 1.14) |
| ADHD | 2.56 (2.46, 2.67) | 2.57 (2.46, 2.67) | 1.38 (1.30, 1.46) | 1.34 (1.26, 1.43) | | 1.42 (1.29, 1.55) |
| Autism & ADHD | 1.66 (1.54, 1.79) | 1.65 (1.53, 1.78) | 1.06 (0.94, 1.20) | 1.10 (0.97, 1.26) | | 0.95 (0.78, 1.16) |
| **Males** |  |  |  |  | |  |
| Control peers | 1.00 | 1.00 | 1.00 | 1.00 | | 1.00 |
| Autism | 0.51 (0.45, 0.57) | 0.49 (0.44, 0.56) | 0.80 (0.55, 1.15) | 0.41 (0.15, 1.09) | | 0.93 (0.63, 1.37) |
| ADHD | 1.67 (1.59, 1.75) | 1.67 (1.59, 1.75) | 1.32 (1.11, 1.58) | 1.72 (1.27, 2.33) | | 1.12 (0.90, 1.39) |
| Autism & ADHD | 0.73 (0.66, 0.82) | 0.73 (0.65, 0.81) | 0.92 (0.64, 1.30) | 0.85 (0.42, 1.72) | | 0.89 (0.59, 1.34) |
|  | | | | |  |  |

**Supplementary Table 28. Fully adjusted* odds ratios, and 95% confidence intervals, of *overall, in-person, and digital SRH visits* across exclusive diagnosis groups, for females and males separately over the study period 2018–2022.**

|  | **Overall visits** | **In-person visits** | **Digital visits** | **Video** | **chat** |
| --- | --- | --- | --- | --- | --- |
|  | **Odds ratio**  **(95% CI)** | **Odds ratio**  **(95% CI)** | **Odds ratio**  **(95% CI)** | **Odds ratio**  **(95% CI)** | **Odds ratio**  **(95% CI)** |
| **Females** |  |  |  |  |  |
| Control peers | 1.00 | 1.00 | 1.00 | 1.00 | 1.00 |
| Autism | 0.65 (0.61, 0.70) | 0.65 (0.60, 0.69) | 0.77 (0.68, 0.86) | 0.70 (0.61, 0.79) | 0.86 (0.72, 1.03) |
| ADHD | 1.99 (1.91, 2.09) | 2.00 (1.91, 2.09) | 1.18 (1.11, 1.26) | 1.15 (1.08, 1.23) | 1.24 (1.13, 1.36) |
| Autism & ADHD | 1.18 (1.09, 1.28) | 1.18 (1.09, 1.28) | 0.87 (0.77, 0.98) | 0.91 (0.80, 1.04) | 0.76 (0.62, 0.95) |
| **Males** |  |  |  |  |  |
| Control peers | 1.00 | 1.00 | 1.00 | 1.00 | 1.00 |
| Autism | 0.46 (0.41, 0.52) | 0.45 (0.39, 0.51) | 0.79 (0.54, 1.15) | 0.30 (0.10, 0.94) | 0.96 (0.65, 1.42) |
| ADHD | 1.45 (1.38, 1.53) | 1.45 (1.38, 1.53) | 1.25 (1.04, 1.51) | 1.51 (1.10, 2.07) | 1.10 (0.88, 1.37) |
| Autism & ADHD | 0.64 (0.58, 0.72) | 0.64 (0.57, 0.71) | 0.87 (0.61, 1.25) | 0.70 (0.33, 1.48) | 0.89 (0.59, 1.34) |

*Adjusted for birth year and birth year squared and sociodemographic predictors (migrant background, parental education, and household income)

**Supplementary Table 29. Birth year and birth year squared adjusted period prevalence and 95% confidence intervals, of *overall, in-person, and digital SRH visits* across exclusive diagnosis groups, for females and males separately over the study period 2018–2022.**

|  | **Overall visits** | **In-person visits** | **Digital visits** | **Video** | **Chat** |
| --- | --- | --- | --- | --- | --- |
|  | **Period prevalence (95% CI)** | **Period prevalence (95% CI)** | **Period prevalence (95% CI)** | **Period prevalence (95% CI)** | **Period prevalence (95% CI)** |
| **Females** |  |  |  |  |  |
| Control peers | 27.1 (26.9, 27.4) | 26.7 (26.5, 27.0) | 3.2 (3.1, 3.3) | 2.5 (2.4, 2.6) | 1.2 (1.2, 1.3) |
| Autism | 25.4 (24.3, 26.4) | 24.8 (23.8, 25.9) | 2.9 (2.6, 3.2) | 2.1 (1.8, 2.3) | 1.1 (0.7, 1.7) |
| ADHD | 44.6 (43.8, 45.4) | 44.1 (43.3, 45.0) | 4.3 (4.1, 4.6) | 3.3 (3.1, 3.5) | 3.3 (2.5, 4.4) |
| Autism & ADHD | 36.2 (34.8, 37.6) | 35.6 (34.2, 37.1) | 3.4 (3.0, 3.8) | 2.7 (2.4, 3.1) | 1.1 (0.7, 1.7) |
| **Males** |  |  |  |  |  |
| Control peers | 4.4 (4.3, 4.6) | 4.2 (4.1, 4.4) | 0.3 (0.2, 0.3) | 0.1 (0.0, 0.1) | 0.2 (0.1, 0.2) |
| Autism | 2.4 (2.1, 2.6) | 2.2 (1.9, 2.5) | 0.2 (0.1, 0.3) | 0.0 (0.0, 0.1) | 0.1 (0.1, 0.2) |
| ADHD | 7.0 (6.7, 7.3) | 6.7 (6.4, 7.0) | 0.3 (0.3, 0.4) | 0.1 (0.1, 0.1) | 0.2 (0.2, 0.2) |
| Autism & ADHD | 3.3 (3.0, 3.7) | 3.2 (2.8, 3.5) | 0.2 (0.2, 0.3) | 0.1 (0.0, 0.1) | 0.1 (0.1, 0.2) |

**Supplementary Table 30. Fully adjusted* period prevalence and 95% confidence intervals of *overall, in-person, and digital SRH visits* across exclusive diagnosis groups, for females and males separately over the study period 2018–2022.**

|  | **Overall visits** | **In-person visits** | **Digital visits** | **Video** | **chat** |
| --- | --- | --- | --- | --- | --- |
|  | **Period prevalence (95% CI)** | **Period prevalence (95% CI)** | **Period prevalence (95% CI)** | **Period prevalence (95% CI)** | **Period prevalence (95% CI)** |
| **Females** |  |  |  |  |  |
| Control peers | 33.8 (33.5, 34.1) | 33.3 (33.0, 33.6) | 4.4 (4.3, 4.5) | 3.4 (3.2, 3.5) | 2.0 (1.9, 2.2) |
| Autism | 26.3 (25.2, 27.5) | 25.7 (24.6, 26.8) | 3.5 (3.1, 3.9) | 2.4 (2.1, 2.7) | 1.4 (0.9, 2.1) |
| ADHD | 47.4 (46.5, 48.3) | 46.8 (45.9, 47.7) | 5.1 (4.8, 5.4) | 3.8 (3.6, 4.1) | 4.0 (2.9, 5.4) |
| Autism & ADHD | 37.0 (35.4, 38.5) | 36.4 (34.8, 37.9) | 3.9 (3.5, 4.4) | 3.1 (2.7, 3.5) | 1.0 (0.6, 1.7) |
| **Males** |  |  |  |  |  |
| Control peers | 5.8 (5.7, 6.0) | 5.6 (5.4, 5.7) | 0.3 (0.3, 0.4) | 0.1 (0.1, 0.1) | 0.2 (0.2, 0.2) |
| Autism | 2.8 (2.5, 3.2) | 2.6 (2.3, 3.0) | 0.3 (0.2, 0.4) | 0.0 (0.0, 0.1) | 0.2 (0.1, 0.2) |
| ADHD | 8.1 (7.7, 8.4) | 7.7 (7.4, 8.1) | 0.4 (0.3, 0.5) | 0.1 (0.1, 0.2) | 0.2 (0.2, 0.2) |
| Autism & ADHD | 3.9 (3.5, 4.3) | 3.7 (3.3, 4.1) | 0.3 (0.2, 0.4) | 0.1 (0.0, 0.1) | 0.2 (0.1, 0.2) |

*Adjusted for birth year and birth year squared and sociodemographic predictors (migrant background, parental education, and household income)

**Supplementary Table 31. Adjusted period prevalence and Odds Ratios of *SRH visits with midwives* at the population level across exclusive diagnosis groups over the study period 2018–2022.**

| Midwives | Birth year and birth year squared adjusted |  | Fully adjusted* |  |
| --- | --- | --- | --- | --- |
|  | **Period prevalence (95% CI)** | **OR (95% CI)** | **Period prevalence (95% CI)** | **OR (95% CI)** |
| Females |  |  |  |  |
| Control peers | 25.9 (25.7, 26.2) | 1.00 | 32.4 (32.1, 32.7) | 1.00 |
| Autism | 23.4 (22.4, 24.4) | 0.85 (0.80, 0.91) | 24.1 (23.0, 25.2) | 0.62 (0.57, 0.66) |
| ADHD | 43.1 (42.3, 43.9) | 2.57 (2.47, 2.68) | 45.8 (44.9, 46.7) | 2.00 (1.92, 2.09) |
| Autism & ADHD | 34.2 (32.8, 35.6) | 1.61 (1.49, 1.74) | 34.9 (33.4, 36.4) | 1.14 (1.06, 1.24) |
| Males |  |  |  |  |
| Control peers | 3.8 (3.7, 4.0) | 1.00 | 5.1 (4.9, 5.2) | 1.00 |
| Autism | 1.9 (1.7, 2.2) | 0.48 (0.42, 0.54) | 2.3 (2.0, 2.6) | 0.43 (0.38, 0.49) |
| ADHD | 6.2 (5.9, 6.5) | 1.70 (1.61, 1.78) | 7.2 (6.8, 7.5) | 1.48 (1.40, 1.56) |
| Autism & ADHD | 2.8 (2.5, 3.1) | 0.71 (0.64, 0.80) | 3.3 (2.9, 3.6) | 0.62 (0.55, 0.70) |

*Adjusted for birth year and birth year squared and sociodemographic predictors (migrant background, parental education, and household income)

**Supplementary Table 32. Adjusted period prevalence and Odds Ratios of *SRH visits with physicians* at the population level across exclusive diagnosis groups over the study period 2018–2022.**

| Physicians | Birth year and birth year squared adjusted |  | Fully adjusted* |  |
| --- | --- | --- | --- | --- |
|  | **Period prevalence (95% CI)** | **OR (95% CI)** | **Period prevalence (95% CI)** | **OR (95% CI)** |
| Females |  |  |  |  |
| Control peers | 8.3 (8.2, 8.5) | 1.00 | 10.4 (10.2, 10.6) | 1.00 |
| Autism | 11.6 (10.8, 12.4) | 1.46 (1.35, 1.59) | 12.4 (11.5, 13.3) | 1.22 (1.12, 1.33) |
| ADHD | 14.9 (14.3, 15.4) | 1.97 (1.88, 2.06) | 15.9 (15.3, 16.5) | 1.65 (1.57, 1.73) |
| Autism & ADHD | 14.5 (13.5, 15.6) | 1.91 (1.75, 2.09) | 15.1 (14.1, 16.3) | 1.55 (1.42, 1.70) |
| Males |  |  |  |  |
| Control peers | 1.0 (0.9, 1.0) | 1.00 | 1.2 (1.2, 1.3) | 1.00 |
| Autism | 0.7 (0.5, 0.8) | 0.68 (0.54, 0.85) | 0.8 (0.6, 1.0) | 0.64 (0.51, 0.81) |
| ADHD | 1.3 (1.2, 1.5) | 1.38 (1.24, 1.52) | 1.5 (1.3, 1.7) | 1.22 (1.10, 1.36) |
| Autism & ADHD | 0.7 (0.6, 0.9) | 0.77 (0.62, 0.96) | 0.9 (0.7, 1.1) | 0.70 (0.56, 0.88) |

*Adjusted for birth year and birth year squared and sociodemographic predictors (migrant background, parental education, and household income)

**Supplementary Table 33. Adjusted period prevalence and Odds Ratios of females aged 15-22 having used *contraceptives* (any, LARC, SARC) or *abortion* over the study period 2018–2022.**

|  | Birth year and birth year squared adjusted | | Fully adjusted* | |
| --- | --- | --- | --- | --- |
|  | **Period prevalence (95% CI)** | **OR (95% CI)** | **Period prevalence (95% CI)** | **OR (95% CI)** |
| Any contraceptive |  |  |  |  |
| Control peers | 54.6 (54.3, 54.9) | 1.00 | 59.1 (58.8, 59.5) | 1.00 |
| Autism | 50.1 (48.3, 52.0) | 0.83 (0.77, 0.90) | 46.8 (44.9, 48.7) | 0.59 (0.54, 0.64) |
| ADHD | 72.5 (71.6, 73.5) | 2.21 (2.11, 2.33) | 69.9 (68.9, 71.0) | 1.66 (1.57, 1.75) |
| Autism & ADHD | 63.5 (61.4, 65.6) | 1.45 (1.32, 1.59) | 58.4 (56.3, 60.7) | 0.97 (0.88, 1.07) |
| LARC |  |  |  |  |
| Control peers | 12.3 (12.1, 12.5) | 1.00 | 13.1 (12.8, 13.3) | 1.00 |
| Autism | 13.8 (12.6, 15.1) | 1.14 (1.02, 1.27) | 12.9 (11.8, 14.2) | 0.99 (0.89, 1.11) |
| ADHD | 23.5 (22.6, 24.4) | 2.20 (2.09, 2.32) | 22.0 (21.1, 22.9) | 1.89 (1.79, 2.00) |
| Autism & ADHD | 20.4 (18.8, 22.1) | 1.83 (1.65, 2.04) | 18.5 (17.0, 20.2) | 1.52 (1.37, 1.70) |
| SARC |  |  |  |  |
| Control peers | 46.8 (46.5, 47.1) | 1.00 | 51.1 (50.8, 51.5) | 1.00 |
| Autism | 41.1 (39.4, 43.0) | 0.79 (0.73, 0.86) | 38.9 (37.1, 40.8) | 0.59 (0.55, 0.64) |
| ADHD | 58.6 (57.6, 59.6) | 1.62 (1.55, 1.69) | 56.0 (54.9, 57.1) | 1.23 (1.17, 1.29) |
| Autism & ADHD | 50.3 (48.2, 52.5) | 1.15 (1.06, 1.26) | 46.4 (44.3, 48.6) | 0.82 (0.75, 0.90) |
| Abortion |  |  |  |  |
| Control peers | 3.3 (3.2, 3.4) | 1.00 | 3.2 (3.0, 3.3) | 1.00 |
| Autism | 2.9 (2.3, 3.5) | 0.86 (0.69, 1.07) | 2.6 (2.1, 3.2) | 0.82 (0.66, 1.02) |
| ADHD | 8.8 (8.2, 9.4) | 2.83 (2.62, 3.06) | 7.2 (6.7, 7.7) | 2.40 (2.20, 2.61) |
| Autism & ADHD | 5.4 (4.5, 6.4) | 1.67 (1.38, 2.01) | 4.3 (3.6, 5.2) | 1.39 (1.14, 1.69) |

*Adjusted for birth year and birth year squared and sociodemographic predictors (migrant background, parental education, and household income)

**Supplementary Table 34. Visits per 1000 individuals and Incident-Rate Ratios (IRR) *of overall SRH visits* among youth with a youth clinic visit, stratified by sex and across exclusive diagnosis groups, over the study period.**

| **Overall SRH visits** |  | **Birth year and birth year squared adjusted** | |  | **Fully adjusted*** | |
| --- | --- | --- | --- | --- | --- | --- |
|  | **Crude visits among visitors, mean (SD)** | **Adjusted rate per 1000 individuals**  **(95% CI)** | **Incidence rate ratio (95% CI)** |  | **Adjusted rate per 1000 individuals**  **(95% CI)** | **Incidence rate ratio (95% CI)** |
| **Females** |  |  |  |  |  |  |
| Control peers | 2.2 (3.8) | 1757 (1747–1767) | 1.00 |  | 2048 (2036–2059) | 1.00 |
| Autism | 2.0 (4.0) | 1844 (1804–1884) | 1.05 (1.03–1.07) |  | 2079 (2033–2126) | 1.02 (0.99–1.04) |
| ADHD | 4.0 (5.2) | 2260 (2237–2283) | 1.29 (1.27–1.30) |  | 2551 (2525–2578) | 1.25 (1.23–1.26) |
| Autism & ADHD | 2.9 (4.3) | 1987 (1945–2028) | 1.13 (1.11–1.15) |  | 2206 (2158–2253) | 1.08 (1.05–1.10) |
| **Males** |  |  |  |  |  |  |
| Control peers | 0.2 (0.9) | 194 (191–197) | 1.00 |  | 216 (213–220) | 1.00 |
| Autism | 0.1 (0.6) | 135 (121–149) | 0.70 (0.63–0.77) |  | 145 (130–160) | 0.67 (0.60–0.74) |
| ADHD | 0.3 (1.1) | 235 (227–242) | 1.21 (1.17–1.25) |  | 258 (250–267) | 1.19 (1.16–1.23) |
| Autism & ADHD | 0.2 (0.9) | 202 (186–219) | 1.04 (0.96–1.13) |  | 218 (200–237) | 1.01 (0.93–1.10) |
| *Adjusted for birth year and birth year squared and sociodemographic predictors (migrant background, parental education, and household income). | | | | | | |

**Supplementary Table 35. Visits per 1000 individuals and Incident-Rate Ratios (IRR) *of in-person SRH visits* among youth with a youth clinic visit, stratified by sex and across exclusive diagnosis groups, over the study period.**

| **In-person SRH visits** |  | **Birth year and birth year squared adjusted** | |  | **Fully adjusted*** | |
| --- | --- | --- | --- | --- | --- | --- |
|  | **Crude visits among visitors, mean (SD)** | **Adjusted rate per 1000 individuals**  **(95% CI)** | **Incidence rate ratio (95% CI)** |  | **Adjusted rate per 1000 individuals**  **(95% CI)** | **Incidence rate ratio (95% CI)** |
| **Females** |  |  |  |  |  |  |
| Control peers | 2.1 (3.6) | 1649 (1640–1659) | 1.00 |  | 1920 (1908–1931) | 1.00 |
| Autism | 1.9 (3.7) | 1748 (1709–1787) | 1.06 (1.04–1.08) |  | 1966 (1920–2011) | 1.02 (1.00–1.05) |
| ADHD | 3.8 (5.0) | 2158 (2135–2180) | 1.31 (1.30–1.32) |  | 2431 (2405–2457) | 1.27 (1.25–1.28) |
| Autism & ADHD | 2.8 (4.0) | 1892 (1851–1933) | 1.15 (1.12–1.17) |  | 2098 (2051–2144) | 1.09 (1.07–1.12) |
| **Males** |  |  |  |  |  |  |
| Control peers | 0.2 (0.9) | 188 (185–191) | 1.00 |  | 209 (206–213) | 1.00 |
| Autism | 0.1 (0.6) | 128 (114–141) | 0.68 (0.61–0.75) |  | 135 (121–150) | 0.65 (0.58–0.72) |
| ADHD | 0.3 (1.1) | 230 (223–237) | 1.22 (1.19–1.26) |  | 253 (245–262) | 1.21 (1.17–1.25) |
| Autism & ADHD | 0.2 (0.9) | 197 (181–213) | 1.05 (0.96–1.14) |  | 211 (192–230) | 1.01 (0.92–1.10) |
| *Adjusted for birth year and birth year squared and sociodemographic predictors (migrant background, parental education, and household income). | | | | | | |

**Supplementary Table 36. Visits per 1000 individuals and Incident-Rate Ratios (IRR) *of digital SRH visits* among youth with a youth clinic visit, stratified by sex and across exclusive diagnosis groups, over the study period.**

| **Digital SRH visits** |  | **Birth year and birth year squared adjusted** | |  | **Fully adjusted*** | |
| --- | --- | --- | --- | --- | --- | --- |
|  | **Crude visits among visitors, mean (SD)** | **Adjusted rate per 1000 individuals**  **(95% CI)** | **Incidence rate ratio (95% CI)** |  | **Adjusted rate per 1000 individuals**  **(95% CI)** | **Incidence rate ratio (95% CI)** |
| **Females** |  |  |  |  |  |  |
| Control peers | 0.2 (0.7) | 126 (124–129) | 1.00 |  | 149 (147–152) | 1.00 |
| Autism | 0.1 (0.7) | 127 (115–139) | 1.01 (0.91–1.10) |  | 145 (131–159) | 0.97 (0.88–1.07) |
| ADHD | 0.2 (0.8) | 148 (141–154) | 1.17 (1.12–1.22) |  | 170 (163–178) | 1.14 (1.09–1.20) |
| Autism & ADHD | 0.2 (0.7) | 139 (126–153) | 1.10 (1.00–1.21) |  | 152 (136–167) | 1.02 (0.92–1.13) |
| **Males** |  |  |  |  |  |  |
| Control peers | 0.0 (0.1) | 7 (7–8) | 1.00 |  | 8 (8–9) | 1.00 |
| Autism | 0.0 (0.1) | 6 (4–8) | 0.82 (0.57–1.18) |  | 7 (4–9) | 0.81 (0.56–1.17) |
| ADHD | 0.0 (0.1) | 8 (6–9) | 1.07 (0.89–1.29) |  | 9 (7–10) | 1.05 (0.87–1.28) |
| Autism & ADHD | 0.0 (0.1) | 6 (4–8) | 0.78 (0.53–1.13) |  | 6 (4–9) | 0.76 (0.52–1.11) |
| *Adjusted for birth year and birth year squared and sociodemographic predictors (migrant background, parental education, and household income). | | | | | | |

## Overlapping Autism and ADHD groups

**Supplementary Table 37. *Sociodemographic characteristics* of the study population by sex and by overlapping autism and ADHD groups among youth aged 12–22 in Stockholm County, 2018–2022.**

| Diagnosis group | Autism  n=8 252 (3.7%) | ADHD  n=16 602 (7.5%) | Control peers  n=199 228 (90.3%) | Autism  n=12 724 (5.4%) | ADHD  n=23 668 (10.1%) | Control peers  n=203 057 (86.9%) |
| --- | --- | --- | --- | --- | --- | --- |
| Mean visits over study period (SD) | 5.3 (4.7) | 6.1 (5.2) | 5.0 (4.3) | 2.2 (2.4) | 2.3 (2.2) | 2.2 (2.0) |
| Age category* |  |  |  |  |  |  |
| 12-14 | 2 920 (9.0) | 5 224 (7.9) | 85 253 (12.9) | 6 016 (12.7) | 10 065 (11.1) | 84 461 (12.4) |
| 15-19 | 17 051 (52.6) | 33 767 (51.2) | 295 303 (44.7) | 23 771 (50.2) | 46 408 (51.1) | 299 921 (43.9) |
| 20-22 | 12 430 (38.4) | 26 999 (40.9) | 280 191 (42.4) | 17 593 (37.1) | 34 286 (37.8) | 298 200 (43.7) |
| Migrant background |  |  |  |  |  |  |
| Swedish-born parents | 6 811 (82.5) | 13 507 (81.4) | 124 890 (62.7) | 9 870 (77.6) | 18 837 (79.6) | 123 765 (61.0) |
| Foreign-born parents | 807 (9.8) | 1 922 (11.6) | 30 666 (15.4) | 1 792 (14.1) | 3 000 (12.7) | 30 125 (14.8) |
| Foreign-born | 634 (7.7) | 1 173 (7.1) | 43 672 (21.9) | 1 062 (8.3) | 1 831 (7.7) | 49 167 (24.2) |
| Primary education | 1 961 (23.8) | 4 577 (27.6) | 46 874 (23.5) | 3 183 (25.0) | 6 650 (28.1) | 45 695 (22.5) |
| Secondary education | 2 984 (36.2) | 6 290 (37.9) | 62 131 (31.2) | 4 497 (35.3) | 8 678 (36.7) | 60 998 (30.0) |
| Post-secondary education | 3 212 (38.9) | 5 543 (33.4) | 74 222 (37.3) | 4 860 (38.2) | 8 074 (34.1) | 74 638 (36.8) |
| Missing | 95 (1.2) | 192 (1.2) | 16 001 (8.0) | 184 (1.4) | 266 (1.1) | 21 726 (10.7) |
| Primary education | 2 823 (34.2) | 6 699 (40.4) | 57 761 (29.0) | 4 356 (34.2) | 9 454 (39.9) | 56 729 (27.9) |
| Secondary education | 2 881 (34.9) | 5 595 (33.7) | 60 171 (30.2) | 4 309 (33.9) | 7 976 (33.7) | 59 608 (29.4) |
| Post-secondary education | 2 176 (26.4) | 3 573 (21.5) | 57 768 (29.0) | 3 434 (27.0) | 5 195 (21.9) | 58 274 (28.7) |
| Missing | 372 (4.5) | 735 (4.4) | 23 528 (11.8) | 625 (4.9) | 1 043 (4.4) | 28 446 (14.0) |
| Household income |  |  |  |  |  |  |
| Q1 | 1 564 (19.0) | 3 154 (19.0) | 37 290 (18.7) | 2 628 (20.7) | 4 486 (19.0) | 36 636 (18.0) |
| Q2 | 1 980 (24.0) | 3 935 (23.7) | 32 680 (16.4) | 2 998 (23.6) | 5 448 (23.0) | 32 426 (16.0) |
| Q3 | 1 823 (22.1) | 3 400 (20.5) | 32 588 (16.4) | 2 537 (19.9) | 4 879 (20.6) | 32 613 (16.1) |
| Q4 | 1 394 (16.9) | 2 900 (17.5) | 33 779 (17.0) | 1 995 (15.7) | 3 891 (16.4) | 33 762 (16.6) |
| Q5 | 1 045 (12.7) | 2 508 (15.1) | 34 695 (17.4) | 1 502 (11.8) | 3 443 (14.5) | 34 617 (17.0) |
| Missing | 446 (5.4) | 705 (4.2) | 28 196 (14.2) | 1 064 (8.4) | 1 521 (6.4) | 33 003 (16.3) |

* Based on age-category for the most recent year (2022 or latest year when the individual was included in the study population).

**Supplementary Table 38. Sociodemographic characteristics of *SRH service users* by sex and by overlapping autism and ADHD groups among youth aged 12–22 in Stockholm County, 2018–2022.**

|  | Female visitors  87 126 (39%) | | | Male visitors  21 769 (9%) | | |
| --- | --- | --- | --- | --- | --- | --- |
| Diagnosis group (n and % with ≥1 SRH visit 2018-2022) | Autism  3 554 (43.1%) | ADHD  9 724 (58.6%) | Control peers  75 592 (37.9%) | Autism  684 (5.4%) | ADHD  2 754 (11.6%) | Control peers  18 719 (9.2%) |
| Mean visits over study period (SD) | 5.3 (4.7) | 6.1 (5.2) | 5.0 (4.3) | 2.2 (2.4) | 2.3 (2.2) | 2.2 (2.0) |
| Migrant background |  |  |  |  |  |  |
| Swedish-born parents | 3 056 (86.0%) | 8 150 (83.8%) | 57 236 (75.7%) | 551 (80.6%) | 2 119 (76.9%) | 12 120 (64.7%) |
| Foreign-born parents | 251 (7.1%) | 953 (9.8%) | 8 499 (11.2%) | 81 (11.8%) | 397 (14.4%) | 2 905 (15.5%) |
| Foreign-born | 247 (6.9%) | 621 (6.4%) | 9 857 (13.0%) | 52 (7.6%) | 238 (8.6%) | 3 694 (19.7%) |
| Maternal education level |  |  |  |  |  |  |
| Primary education | 903 (25.4%) | 2 845 (29.3%) | 18 138 (24.0%) | 199 (29.1%) | 949 (34.5%) | 4 985 (26.6%) |
| Secondary education | 1 360 (38.3%) | 3 775 (38.8%) | 26 802 (35.5%) | 239 (34.9%) | 967 (35.1%) | 6 068 (32.4%) |
| Post-secondary education | 1 261 (35.5%) | 3 025 (31.1%) | 28 262 (37.4%) | 238 (34.8%) | 805 (29.2%) | 6 290 (33.6%) |
| Missing | 30 (0.8%) | 79 (0.8%) | 2 390 (3.2%) | 8 (1.2%) | 33 (1.2%) | 1 376 (7.4%) |
| Paternal education level |  |  |  |  |  |  |
| Primary education | 1 336 (37.6%) | 4 211 (43.3%) | 24 137 (31.9%) | 279 (40.8%) | 1 270 (46.1%) | 6 144 (32.8%) |
| Secondary education | 1 240 (34.9%) | 3 192 (32.8%) | 24 996 (33.1%) | 215 (31.4%) | 857 (31.1%) | 5 607 (30.0%) |
| Post-secondary education | 838 (23.6%) | 1 928 (19.8%) | 21 163 (28.0%) | 150 (21.9%) | 480 (17.4%) | 4 902 (26.2%) |
| Missing | 140 (3.9%) | 393 (4.0%) | 5 296 (7.0%) | 40 (5.8%) | 147 (5.3%) | 2 066 (11.0%) |
| Household income |  |  |  |  |  |  |
| Q1 | 644 (18.1%) | 1 827 (18.8%) | 10 867 (14.4%) | 161 (23.5%) | 603 (21.9%) | 3 276 (17.5%) |
| Q2 | 891 (25.1%) | 2 394 (24.6%) | 13 734 (18.2%) | 181 (26.5%) | 682 (24.8%) | 3 374 (18.0%) |
| Q3 | 849 (23.9%) | 2 124 (21.8%) | 15 511 (20.5%) | 138 (20.2%) | 580 (21.1%) | 3 483 (18.6%) |
| Q4 | 641 (18.0%) | 1 778 (18.3%) | 16 602 (22.0%) | 113 (16.5%) | 449 (16.3%) | 3 587 (19.2%) |
| Q5 | 498 (14.0%) | 1 548 (15.9%) | 17 008 (22.5%) | 84 (12.3%) | 423 (15.4%) | 3 979 (21.3%) |
| Missing | 31 (0.9%) | 53 (0.5%) | 1 870 (2.5%) | 7 (1.0%) | 17 (0.6%) | 1 020 (5.4%) |

**Supplementary Table 39. Adjusted period prevalence and Odds Ratios of *overall SRH visits* at the population level by overlapping autism and ADHD groups over the entire study period 2018-2022. Error bars represent 95% confidence interval**

|  | Model I. Birth-year | | Model II. Birth-year and comorbidity^*^ | | Model III. Birth-year, comorbidity, and sociodemographic predictors^**^ | |
| --- | --- | --- | --- | --- | --- | --- |
|  | **Period prevalence (95% CI)** | **OR (95% CI)** | **Period prevalence (95% CI)** | **OR (95% CI)** | **Period prevalence (95% CI)** | **OR (95% CI)** |
| Females |  |  |  |  |  |  |
| No autism diagnosis | 48.3 (48.0, 48.6) | 1.00 | 48.6 (48.3, 48.9) | 1.00 | 58.2 (57.9, 58.6) | 1.00 |
| Autism diagnosis | 51.4 (50.0, 52.9) | 1.11 (1.06, 1.17) | 42.8 (41.5, 44.2) | 0.81 (0.77, 0.86) | 45.9 (44.4, 47.4) | 0.66 (0.62, 0.69) |
| No ADHD diagnosis | 46.4 (46.2, 46.7) | 1.00 | 46.4 (46.1, 46.6) | 1.00 | 55.9 (55.5, 56.2) | 1.00 |
| ADHD diagnosis | 73.1 (72.0, 74.2) | 2.36 (2.27, 2.44) | 74.2 (73.1, 75.4) | 2.45 (2.36, 2.54) | 77.7 (76.5, 79.0) | 1.99 (1.91, 2.07) |
| Males |  |  |  |  |  |  |
| No autism diagnosis | 10.0 (9.8, 10.1) | 1.00 | 10.1 (9.9, 10.2) | 1.00 | 11.5 (11.3, 11.6) | 1.00 |
| Autism diagnosis | 6.1 (5.6, 6.5) | 0.58 (0.54, 0.63) | 5.0 (4.6, 5.4) | 0.47 (0.44, 0.51) | 5.6 (5.1, 6.0) | 0.45 (0.42, 0.50) |
| No ADHD diagnosis | 9.4 (9.3, 9.6) | 1.00 | 9.3 (9.2, 9.5) | 1.00 | 10.6 (10.5, 10.8) | 1.00 |
| ADHD diagnosis | 13.0 (12.5, 13.5) | 1.44 (1.38, 1.50) | 14.4 (13.9, 15.0) | 1.64 (1.57, 1.72) | 14.8 (14.2, 15.3) | 1.46 (1.39, 1.53) |

*Autism and ADHD estimates are mutually adjusted for comorbidity. **Sociodemographic predictors include migrant background, parental education, and household income.

**40. Adjusted period prevalence and Odds Ratios of *in-person SRH visits* at the population level by overlapping autism and ADHD groups over the entire study period, 2018–2022.**

| No autism diagnosis | 47.5 (47.2, 47.7) | 1.00 | 47.8 (47.5, 48.1) | 1.00 | 57.3 (57.0, 57.6) | 1.00 |
| --- | --- | --- | --- | --- | --- | --- |
| Autism diagnosis | 50.4 (49.0, 51.8) | 1.11 (1.05, 1.16) | 41.9 (40.5, 43.2) | 0.81 (0.77, 0.85) | 44.8 (43.4, 46.3) | 0.65 (0.62, 0.69) |
| No ADHD diagnosis | 45.6 (45.4, 45.9) | 1.00 | 45.6 (45.3, 45.8) | 1.00 | 54.9 (54.6, 55.2) | 1.00 |
| ADHD diagnosis | 72.2 (71.1, 73.3) | 2.36 (2.27, 2.44) | 73.3 (72.2, 74.5) | 2.45 (2.36, 2.54) | 76.7 (75.5, 78.0) | 1.99 (1.91, 2.07) |
| Males |  |  |  |  |  |  |
| No autism diagnosis | 9.7 (9.6, 9.9) | 1.00 | 9.8 (9.7, 10.0) | 1.00 | 11.2 (11.1, 11.4) | 1.00 |
| Autism diagnosis | 5.9 (5.4, 6.3) | 0.58 (0.53, 0.62) | 4.8 (4.5, 5.2) | 0.46 (0.43, 0.50) | 5.3 (4.9, 5.8) | 0.45 (0.41, 0.49) |
| No ADHD diagnosis | 9.2 (9.1, 9.3) | 1.00 | 9.1 (9.0, 9.2) | 1.00 | 10.4 (10.3, 10.6) | 1.00 |
| ADHD diagnosis | 12.7 (12.3, 13.2) | 1.65 (1.57, 1.73) | 14.2 (13.6, 14.7) | 1.46 (1.39, 1.53) | 14.5 (13.9, 15.1) | 1.46 (1.39, 1.53) |

*Autism and ADHD estimates are mutually adjusted for comorbidity. **Sociodemographic predictors include migrant background, parental education, and household income.

**41. Period prevalence and Odds Ratios of *digital SRH visits* at the population level by overlapping autism and ADHD groups over the entire study period, 2018–2022.**

| No autism diagnosis | 7.7 (7.5, 7.8) | 1.00 | 7.7 (7.6, 7.8) | 1.00 | 9.3 (9.1, 9.4) | 1.00 |
| --- | --- | --- | --- | --- | --- | --- |
| Autism diagnosis | 7.5 (6.9, 8.0) | 0.97 (0.90, 1.06) | 6.7 (6.2, 7.2) | 0.86 (0.79, 0.94) | 7.4 (6.8, 7.9) | 0.77 (0.71, 0.84) |
| No ADHD diagnosis | 7.5 (7.3, 7.6) | 1.00 | 7.4 (7.3, 7.6) | 1.00 | 9.0 (8.9, 9.2) | 1.00 |
| ADHD diagnosis | 9.8 (9.3, 10.2) | 1.35 (1.28, 1.42) | 10.0 (9.5, 10.4) | 1.39 (1.31, 1.46) | 10.7 (10.2, 11.2) | 1.21 (1.14, 1.28) |
| Males |  |  |  |  |  |  |
| No autism diagnosis | 0.6 (0.5, 0.6) | 1.00 | 0.6 (0.5, 0.6) | 1.00 | 0.7 (0.6, 0.7) | 1.00 |
| Autism diagnosis | 0.5 (0.4, 0.6) | 0.84 (0.65, 1.09) | 0.4 (0.3, 0.5) | 0.75 (0.57, 0.98) | 0.5 (0.4, 0.6) | 0.74 (0.57, 0.98) |
| No ADHD diagnosis | 0.5 (0.5, 0.6) | 1.00 | 0.5 (0.5, 0.6) | 1.00 | 0.6 (0.6, 0.7) | 1.00 |
| ADHD diagnosis | 0.7 (0.6, 0.8) | 1.25 (1.06, 1.47) | 0.7 (0.6, 0.8) | 1.32 (1.12, 1.56) | 0.8 (0.7, 0.9) | 1.25 (1.05, 1.49) |

*Autism and ADHD estimates are mutually adjusted for comorbidity. **Sociodemographic predictors include migrant background, parental education, and household income.

**Supplementary Table 42. Adjusted period prevalence and Odds Ratios of females aged 15-22 having used *contraceptives* (any, LARC, SARC) or *abortion* over the study period 2018–2022 by overlapping autism and ADHD groups (N=174 870).**

| Model I* | Model I. Birth-year | | Model II. Birth-year and comorbidity^*^ | | Model III. Birth-year, comorbidity, and sociodemographic predictors^**^ | |
| --- | --- | --- | --- | --- | --- | --- |
|  | **Period prevalence (95% CI)** | **OR (95% CI)** | **Period prevalence (95% CI)** | **OR (95% CI)** | **Period prevalence (95% CI)** | **OR (95% CI)** |
| Any contraceptive |  |  |  |  |  |  |
| No autism diagnosis | 55.9 (55.6, 56.2) | 1.00 | 56.0 (55.7, 56.2) | 1.00 | 60.2 (59.9, 60.5) | 1.00 |
| Autism diagnosis | 56.3 (54.9, 57.7) | 1.02 (0.96, 1.08) | 49.7 (48.3, 51.2) | 0.78 (0.73, 0.83) | 48.2 (46.7, 49.7) | 0.60 (0.56, 0.64) |
| No ADHD diagnosis | 54.6 (54.4, 54.9) | 1.00 | 54.5 (54.3, 54.8) | 1.00 | 58.8 (58.5, 59.1) | 1.00 |
| ADHD diagnosis | 71.3 (70.4, 72.2) | 2.17 (2.07, 2.27) | 72.1 (71.2, 73.0) | 1.69 (1.61, 1.78) | 70.1 (69.1, 71.0) | 1.69 (1.61, 1.78) |
| LARC |  |  |  |  |  |  |
| No autism diagnosis | 13.3 (13.2, 13.5) | 1.00 | 13.2 (13.1, 13.4) | 1.00 | 14.0 (13.8, 14.2) | 1.00 |
| Autism diagnosis | 17.2 (16.2, 18.3) | 1.35 (1.25, 1.45) | 13.2 (12.3, 14.1) | 0.99 (0.92, 1.08) | 12.9 (12.1, 13.9) | 0.91 (0.84, 0.99) |
| No ADHD diagnosis | 12.6 (12.4, 12.8) | 1.00 | 12.6 (12.4, 12.8) | 1.00 | 13.3 (13.1, 13.5) | 1.00 |
| ADHD diagnosis | 23.5 (22.7, 24.3) | 2.14 (2.03, 2.24) | 23.5 (22.7, 24.3) | 1.86 (1.77, 1.96) | 22.2 (21.4, 23.0) | 1.86 (1.77, 1.96) |
| SARC |  |  |  |  |  |  |
| No autism diagnosis | 47.8 (47.5, 48.0) | 1.00 | 47.8 (47.5, 48.1) | 1.00 | 51.8 (51.4, 52.1) | 1.00 |
| Autism diagnosis | 45.5 (44.1, 46.9) | 0.91 (0.86, 0.97) | 41.3 (39.9, 42.8) | 0.77 (0.72, 0.81) | 40.7 (39.3, 42.2) | 0.63 (0.59, 0.67) |
| No ADHD diagnosis | 46.9 (46.6, 47.1) | 1.00 | 46.8 (46.5, 47.0) | 1.00 | 50.8 (50.5, 51.1) | 1.00 |
| ADHD diagnosis | 57.6 (56.6, 58.5) | 1.62 (1.55, 1.69) | 58.6 (57.6, 59.5) | 1.27 (1.22, 1.33) | 56.5 (55.5, 57.5) | 1.27 (1.22, 1.33) |
| Abortion |  |  |  |  |  |  |
| No autism diagnosis | 3.8 (3.7, 3.9) | 1.00 | 3.7 (3.6, 3.8) | 1.00 | 3.5 (3.4, 3.7) | 1.00 |
| Autism diagnosis | 4.1 (3.6, 4.7) | 1.09 (0.94, 1.25) | 2.6 (2.3, 3.0) | 0.70 (0.61, 0.81) | 2.5 (2.1, 2.8) | 0.69 (0.59, 0.80) |
| No ADHD diagnosis | 3.4 (3.3, 3.5) | 1.00 | 3.4 (3.3, 3.5) | 1.00 | 3.2 (3.1, 3.3) | 1.00 |
| ADHD diagnosis | 8.4 (7.9, 9.0) | 2.75 (2.55, 2.97) | 8.8 (8.2, 9.4) | 2.34 (2.16, 2.54) | 7.2 (6.7, 7.7) | 2.34 (2.16, 2.54) |

*Autism and ADHD estimates are mutually adjusted for comorbidity. **Sociodemographic predictors include migrant background, parental education, and household income.

**Supplementary Table 43. Adjusted period prevalence and Odds Ratios of visit with midwives by sex and overlapping autism and ADHD groups over the entire study period, 2018–2022.**

| No autism diagnosis | 26.4 (26.1, 26.8) | 1.00 | 26.3 (26.0, 26.7) | 1.00 | 33.8 (33.5, 34.2) | 1.00 |
| --- | --- | --- | --- | --- | --- | --- |
| Autism diagnosis | 27.4 (26.6, 28.3) | 1.06 (1.01, 1.12) | 22.4 (21.6, 23.2) | 0.78 (0.74, 0.82) | 25.5 (24.6, 26.4) | 0.62 (0.59, 0.66) |
| No ADHD diagnosis | 25.4 (25.0, 25.7) | 1.00 | 25.3 (25.0, 25.6) | 1.00 | 32.5 (32.2, 32.9) | 1.00 |
| ADHD diagnosis | 40.4 (39.6, 41.2) | 2.35 (2.26, 2.43) | 41.1 (40.3, 42.0) | 2.46 (2.37, 2.55) | 46.1 (45.2, 46.9) | 2.00 (1.92, 2.08) |
| Males |  |  |  |  |  |  |
| No autism diagnosis | 4.3 (4.1, 4.5) | 1.00 | 4.3 (4.1, 4.5) | 1.00 | 5.8 (5.6, 6.0) | 1.00 |
| Autism diagnosis | 2.5 (2.3, 2.7) | 0.56 (0.51, 0.61) | 2.0 (1.8, 2.2) | 0.45 (0.41, 0.49) | 2.6 (2.4, 2.9) | 0.43 (0.39, 0.47) |
| No ADHD diagnosis | 4.0 (3.8, 4.2) | 1.00 | 3.9 (3.7, 4.1) | 1.00 | 5.3 (5.1, 5.5) | 1.00 |
| ADHD diagnosis | 5.6 (5.3, 6.0) | 1.45 (1.39, 1.52) | 6.2 (5.9, 6.6) | 1.67 (1.59, 1.75) | 7.5 (7.1, 7.9) | 1.48 (1.41, 1.56) |

*Autism and ADHD estimates are mutually adjusted for comorbidity. **Sociodemographic predictors include migrant background, parental education, and household income.

**Supplementary Table 44. Adjusted period prevalence and Odds Ratios of visit with physicians by sex and overlapping autism and ADHD groups over the entire study period, 2018–2022.**

| No autism diagnosis | 8.8 (8.6, 8.9) | 1.00 | 8.7 (8.6, 8.9) | 1.00 | 11.0 (10.9, 11.2) | 1.00 |
| --- | --- | --- | --- | --- | --- | --- |
| Autism diagnosis | 13.0 (12.3, 13.6) | 1.57 (1.48, 1.67) | 10.5 (10.0, 11.1) | 1.24 (1.16, 1.32) | 12.0 (11.4, 12.7) | 1.11 (1.04, 1.18) |
| No ADHD diagnosis | 8.4 (8.3, 8.6) | 1.00 | 8.4 (8.3, 8.6) | 1.00 | 10.7 (10.5, 10.9) | 1.00 |
| ADHD diagnosis | 15.0 (14.5, 15.5) | 1.96 (1.88, 2.04) | 14.5 (14.0, 15.1) | 1.88 (1.81, 1.97) | 16.1 (15.5, 16.6) | 1.62 (1.55, 1.69) |
| Males |  |  |  |  |  |  |
| No autism diagnosis | 1.0 (1.0, 1.1) | 1.00 | 1.0 (1.0, 1.1) | 1.00 | 1.4 (1.3, 1.4) | 1.00 |
| Autism diagnosis | 0.7 (0.6, 0.9) | 0.70 (0.60, 0.82) | 0.6 (0.5, 0.8) | 0.62 (0.52, 0.73) | 0.8 (0.7, 1.0) | 0.61 (0.51, 0.72) |
| No ADHD diagnosis | 1.0 (0.9, 1.1) | 1.00 | 1.0 (0.9, 1.1) | 1.00 | 1.3 (1.2, 1.4) | 1.00 |
| ADHD diagnosis | 1.2 (1.1, 1.4) | 1.24 (1.13, 1.36) | 1.3 (1.2, 1.5) | 1.35 (1.23, 1.49) | 1.6 (1.4, 1.7) | 1.22 (1.10, 1.35) |

*Autism and ADHD estimates are mutually adjusted for comorbidity. **Sociodemographic predictors include migrant background, parental education, and household income.

**Supplementary Table** 45. Visits per 1000 individuals and Incident-Rate Ratios (IRR) of *overall SRH visits* by sex and by overlapping autism and ADHD groups over the study period (95% CI).

|  | Model I. Birth-year | | Model II. Birth-year and comorbidity^*^ | | Model III. Birth-year, comorbidity, and sociodemographic predictors^**^ | |
| --- | --- | --- | --- | --- | --- | --- |
|  | **Visits per 1000 individuals (95% CI)** | **IRR (95% CI)** | **Visits per 1000 individuals (95% CI)** | **IRR (95% CI)** | **Visits per 1000 individuals (95% CI)** | **IRR (95% CI)** |
| Females |  |  |  |  |  |  |
| No autism diagnosis | 1854 (1843–1865) | 1.00 | 1848 (1837–1859) | 1.00 | 2135 (2123–2147) | 1.00 |
| Autism diagnosis | 1961 (1930–1991) | 1.06 (1.04–1.07) | 1782 (1753–1810) | 0.96 (0.95–0.98) | 2016 (1983–2049) | 0.94 (0.93–0.96) |
| No ADHD diagnosis | 1809 (1798–1819) | 1.00 | 1808 (1797–1818) | 1.00 | 2086 (2074–2098) | 1.00 |
| ADHD diagnosis | 2251 (2230–2272) | 1.18 (1.15–1.22) | 2262 (2240–2284) | 1.21 (1.17–1.24) | 2547 (2522–2572) | 1.20 (1.16–1.24) |
| Males |  |  |  |  |  |  |
| No autism diagnosis | 201 (198–203) | 1.00 | 200 (198–203) | 1.00 | 224 (220–227) | 1.00 |
| Autism diagnosis | 174 (162–185) | 0.87 (0.81–0.93) | 160 (149–170) | 0.80 (0.74–0.85) | 174 (161–186) | 0.78 (0.72–0.83) |
| No ADHD diagnosis | 196 (193–198) | 1.00 | 195 (192–197) | 1.00 | 216 (213–219) | 1.00 |
| ADHD diagnosis | 231 (224–238) | 1.18 (1.15–1.22) | 235 (228–242) | 1.21 (1.17–1.24) | 259 (251–267) | 1.20 (1.16–1.24) |

*Autism and ADHD estimates are mutually adjusted for comorbidity. **Sociodemographic predictors include migrant background, parental education, and household income.

**Supplementary Table 46. Visits per 1000 individuals and Incident-Rate Ratios (IRR) of *in-person SRH visits* by sex and by overlapping autism and ADHD groups over the study period (95% CI).**

|  | Model I. Birth-year | | Model II. Birth-year and comorbidity^*^ | | Model III. Birth-year, comorbidity, and sociodemographic predictors^**^ | |
| --- | --- | --- | --- | --- | --- | --- |
|  | **Visits per 1000 individuals (95% CI)** | **IRR (95% CI)** | **Visits per 1000 individuals (95% CI)** | **IRR (95% CI)** | **Visits per 1000 individuals (95% CI)** | **IRR (95% CI)** |
| Females |  |  |  |  |  |  |
| No autism diagnosis | 1729 (1719–1739) | 1.00 | 1724 (1714–1734) | 1.00 | 1992 (1980–2004) | 1.00 |
| Autism diagnosis | 1850 (1821–1880) | 1.07 (1.05–1.09) | 1668 (1640–1695) | 0.97 (0.95–0.98) | 1887 (1855–1919) | 0.95 (0.93–0.96) |
| No ADHD diagnosis | 1685 (1675–1695) | 1.00 | 1684 (1674–1694) | 1.00 | 1943 (1931–1954) | 1.00 |
| ADHD diagnosis | 2136 (2115–2157) | 1.27 (1.26–1.28) | 2145 (2124–2167) | 1.27 (1.26–1.29) | 2414 (2389–2438) | 1.24 (1.23–1.25) |
| Males |  |  |  |  |  |  |
| No autism diagnosis | 194 (191–197) | 1.00 | 194 (191–197) | 1.00 | 216 (213–220) | 1.00 |
| Autism diagnosis | 167 (156–178) | 0.86 (0.80–0.92) | 152 (142–163) | 0.78 (0.73–0.84) | 164 (152–176) | 0.76 (0.70–0.82) |
| No ADHD diagnosis | 189 (186–192) | 1.00 | 188 (185–191) | 1.00 | 208 (205–212) | 1.00 |
| ADHD diagnosis | 226 (219–233) | 1.20 (1.16–1.23) | 230 (223–237) | 1.23 (1.19–1.26) | 254 (245–262) | 1.22 (1.18–1.26) |

*Autism and ADHD estimates are mutually adjusted for comorbidity. **Sociodemographic predictors include migrant background, parental education, and household income.

**Supplementary Table 47. Visits per 1000 individuals and Incident-Rate Ratios (IRR) of *digital SRH visits* by sex and by overlapping autism and ADHD groups over the study period (95% CI).**

|  | Model I. Birth-year | | Model II. Birth-year and comorbidity^*^ | | Model III. Birth-year, comorbidity, and sociodemographic predictors^**^ | |
| --- | --- | --- | --- | --- | --- | --- |
|  | **Visits per 1000 individuals (95% CI)** | **IRR (95% CI)** | **Visits per 1000 individuals (95% CI)** | **IRR (95% CI)** | **Visits per 1000 individuals (95% CI)** | **IRR (95% CI)** |
| Females |  |  |  |  |  |  |
| No autism diagnosis | 127 (125–129) | 1.00 | 127 (125–129) | 1.00 | 150 (147–152) | 1.00 |
| Autism diagnosis | 133 (124–142) | 1.05 (0.98–1.13) | 126 (117–134) | 0.99 (0.92–1.06) | 143 (132–153) | 0.95 (0.89–1.02) |
| No ADHD diagnosis | 125 (123–127) | 1.00 | 125 (123–127) | 1.00 | 147 (145–150) | 1.00 |
| ADHD diagnosis | 146 (140–152) | 1.17 (1.12–1.22) | 146 (140–152) | 1.17 (1.12–1.22) | 168 (161–175) | 1.14 (1.09–1.19) |
| Males |  |  |  |  |  |  |
| No autism diagnosis | 7 (7–8) | 1.00 | 7 (7–8) | 1.00 | 8 (8–9) | 1.00 |
| Autism diagnosis | 6 (4–7) | 0.81 (0.62–1.05) | 6 (4–7) | 0.79 (0.60–1.04) | 6 (5–8) | 0.78 (0.59–1.02) |
| No ADHD diagnosis | 7 (7–8) | 1.00 | 7 (7–7) | 1.00 | 8 (8–9) | 1.00 |
| ADHD diagnosis | 7 (6–8) | 1.01 (0.85–1.20) | 7 (6–9) | 1.05 (0.88–1.26) | 8 (7–10) | 1.03 (0.86–1.24) |

*Autism and ADHD estimates are mutually adjusted for comorbidity. **Sociodemographic predictors include migrant background, parental education, and household income.
